# Permutation tests for experimental data

**DOI:** 10.1007/s10683-023-09799-6

**Published:** 2023-04-01

**Authors:** Charles A. Holt, Sean P. Sullivan

**Affiliations:** 1 Department of Economics, University of Virginia, Charlottesville, VA 22903 USA; 2 University of Iowa College of Law, Iowa City, IA 52241 USA

**Keywords:** Permutation test, Randomization test, Experimental economics, Nonparametric, C12, C14, C90

## Abstract

This article surveys the use of nonparametric permutation tests for analyzing experimental data. The permutation approach, which involves randomizing or permuting features of the observed data, is a flexible way to draw statistical inferences in common experimental settings. It is particularly valuable when few independent observations are available, a frequent occurrence in controlled experiments in economics and other social sciences. The permutation method constitutes a comprehensive approach to statistical inference. In two-treatment testing, permutation concepts underlie popular rank-based tests, like the Wilcoxon and Mann–Whitney tests. But permutation reasoning is not limited to ordinal contexts. Analogous tests can be constructed from the permutation of measured observations—as opposed to rank-transformed observations—and we argue that these tests should often be preferred. Permutation tests can also be used with multiple treatments, with ordered hypothesized effects, and with complex data-structures, such as hypothesis testing in the presence of nuisance variables. Drawing examples from the experimental economics literature, we illustrate how permutation testing solves common challenges. Our aim is to help experimenters move beyond the handful of overused tests in play today and to instead see permutation testing as a flexible framework for statistical inference.

**Supplementary Information:**

The online version contains supplementary material available at 10.1007/s10683-023-09799-6.

## Introduction and motivation

In economics and other social sciences, data from laboratory and field experiments present two common challenges for statistical inference. The first is interdependence. Markets and other group interactions create dependence relationships between observational units. The second challenge is small sample sizes. The costs of recruiting and incentivizing subjects to participate in research experiments often force experimenters to settle for fewer observations than we might like. These costs were magnified during the recent Covid-19 lockdowns that required interactive experiments to be run online, with a significant fraction of Zoom sessions being interrupted by subjects leaving the meeting or experiencing connectivity issues. Sample sizes can also be small in natural experiments, especially where there is little exogenous dispersion of treatment conditions.^1^ While experimenters have never let these challenges stand in the way of useful research, neither have we grappled as seriously as we might with the question of how best to tailor statistical inference to our needs.

A common but conservative approach to addressing the interdependence problem is to perform statistical inference on a summary measure of behavior that can plausibly be interpreted as independent within the overall design of the experiment. Suppose an experiment assigns separate groups of subjects to 8 sessions, with each session involving 10 replications of a simulated market. This approach would compute a single average efficiency measure for each session, yielding a final sample of 8 independent observations for the whole experiment. The argument for aggregating so much of the data is not that economists cannot make progress with models of lower-level individual interactions; economics is replete with such tools. The problem is that the assumptions used to motivate sophisticated empirical models can lack credibility in an experimental context, especially when things like rationality and perfect foresight assumptions are the very things being tested—not assumed—in the study.

Aggregating lower-level observations can mitigate dependence problems but only by exacerbating the second challenge of experimental analysis: small sample sizes. When the experimenter is limited to few data points—perhaps six independent observations for an entire study—common tests struggle to justify statistical inference. Small sample sizes make it difficult to assess the distributional assumptions that parametric tests rely upon. Even more so, small sample sizes will usually preclude reliance on tests that use limit theorems to motivate their null distributions. To address these problems, experimenters turn to nonparametric tests that sacrifice statistical power for validity under a range of distributional conditions.

The use of nonparametric tests is now common in experimental research, but the selection of tests often seems to be driven more by familiarity (and perhaps literature lock-in) than by the properties of the tests themselves. This mirrors how experimental methods are taught. If you ask a colleague how they introduce nonparametric testing in their graduate classes, the response will probably be that they instruct by example, presenting specific applications from papers as they arise. This approach has the advantage of introducing students to tests that are appropriate for common data patterns. But it has the disadvantage of obscuring relationships between different tests and the tradeoffs between them. Little is gained by directing students to textbooks for these additional details. Traditional statistics texts cover a wide array of techniques, beginning (and, for busy graduate students and experimentalists, often ending) with tests of limited relevance to the numerical, multi-dimensional data frequently encountered in experiments.

From an experimenter’s perspective, a better resource is something like Sidney Siegel’s (1956) classic: *Non-parametric Statistics for the Behavioral Sciences*.^2^ Siegel’s presentations are clear, insightful, and laden with intuition. Even better, Siegel draws examples from behavioral psychology and economics experiments, so his presentations of statistical methods build upon and inform experimental design skills. While much of Siegel’s text has stood the test of time, the past 60 years have witnessed large advances in computing power and the consequent enabling of new capabilities in computational statistical analysis. These new capabilities deserve an equally accessible introduction to the field.

This article follows Siegel’s lead in emphasizing intuition and relevant examples while introducing a family of permutation tests that can be used to solve experimental data analysis challenges. The idea behind these tests is to take seriously the experimental design that generated the data, relying on knowledge of experimental randomization and the null hypothesis of no treatment effect to construct statistical tests customized to individual applications. Common nonparametric tests like the Mann–Whitney and Wilcoxon tests are special cases of the general approach we describe, derived by applying permutation methods to ordinal data or to rank-transformed versions of interval measurements. In categorical data analysis, Fisher’s exact test shares a similar motivation (Fisher, 1935). The methodology of permutation testing will be familiar to those who have experience with bootstrap tests (Efron & Tibshirani, 1993), but with one important difference: whereas the theory behind bootstrap inference is based on random resampling *with replacement* from the observed data, the theory behind permutation inference is based on exhaustive permutation of the observed data or random sampling *without replacement*. Manly (2007) discusses the implementation details and strengths of each approach. For our purposes, it is enough to note that permutation methods work well for small samples while the attractive properties of bootstrap methods are usually asymptotic.

Beyond exposing the intuitive methodology of permutation testing, we also offer two comments on the relative attractiveness of different permutation tests. First, rank-based tests are overused today. Currently obscure tests based on (untransformed) interval data present intuitive and possibly more powerful bases for statistical inference. Second, an underappreciated property of all permutation methods is the ability of these tests to accommodate data problems like interdependence, secondary nuisance variables, and other strata that can easily and intuitively be incorporated as permutation constraints.

Using data from various experiments to provide concrete examples, this paper illustrates these and other properties of permutation tests in the analysis of experimental data. We begin in Part 2 with an introduction to sample permutation testing for $k = 2$ independent samples, comparing the now common Mann–Whitney test to the simpler and potentially more powerful Pitman permutation test. Part 3 does the same for data composed of matched pairs of observations. Parts 4–5 generalize two-sample intuition to cases involving $k > 2$ samples. Part 6 introduces stratified permutation methods. Finally, Part 7 briefly discusses the use of permutation methods for correlation and linear effects models. Table 1 summarizes the tests we present and the relationships between them.

**Table 1:** Reference table

Samples compared	Relationship between samples	Permutated feature of collected sample	
		Rank-transformed observations	Measured observations
*k* = 2	Independent	**Mann–Whitney Test** Part 2.2	**Pitman Test** Part 2.1
*k* = 2	Dependent (matched pairs)	**Wilcoxon Test** Part 3.2	**Fisher Test** Part 3.1
*k* > 2	Independent (unordered hypothesis)	**Kruskal-Wallace Test** Part 4.2	**Permutation ***F Test* Part 4.1
*k* > 2	Independent (ordered hypothesis)	**Jonckheere–Terpstra Test** Part 5.1	**Directional Difference Test** Part 5.2
*k* = 2	Dependent	**Stratified Mann–Whitney Test** Part 6.1	**Stratified Pitman Test** Part 6.1
*k* > 2	Dependent	**Friedman Test** Part 6.2	**Stratified Permutation ***F*** Test** Part 6.2
*k* ≥ 2	Associated	**Ordinal Association Test** Part 7.1	**Correlation, Regression Test** Parts 7.1, 7.2

## Permutation tests for *k* = 2 independent samples

The most fundamental statistical test for experimental work is the comparison of averages between unmatched samples. This situation is typical of experiments that draw subjects from a common pool, exposing each person to a single treatment. When contrasting measurements collected under one treatment, $\left\{ {x_{1} , \ldots ,x_{n} } \right\} \sim F_{x} ,$ against those collected under another treatment $\left\{ {y_{1} , \ldots ,y_{m} } \right\} \sim F_{y}$, the null hypothesis of no treatment effect corresponds to a situation in which measurements from both samples are independent and identically distributed draws from the same underlying distribution: $F_{x} = F_{y} = F$.

Appropriate alternative hypotheses are derived from context and theory, as illustrated in the examples below. For simplicity, we confine our discussion to “shift” models in which distributions are assumed to differ in a measure of central tendency if at all. This assumption will often be plausible in the experimental context—especially where the only difference between observations is random assignment to a particular treatment—but is not innocuous and should not be ignored. Permutation tests can have power to detect not just differences in central tendency but also differences in distribution shape and variability. The assumption of a shift model justifies attributing rejection of the null hypothesis to a difference in central tendency. When distributions may differ in not just location but also variance or shape, permutation tests with alternative hypothesis limited to locational shift may fail to control the probability of Type I error. This behavior is discussed and illustrated by Boik (1987), Romano (1990), Hayes (2000), and others. One solution is to adopt a more general alternative hypothesis. Another solution may be to control Type I error rates through modified tests (Feltovich, 2003) or testing algorithms (Chung & Romano, 2016; Neuhäuser & Manly, 2004). For clarity, and to focus on the intuition behind permutation testing, we maintain the shift-model assumption throughout our discussion.

To keep things concrete, consider a classic two-treatment experiment that Caginalp et al. (1998) used to evaluate factors that cause asset share prices to deviate from fundamental values of those shares. Each of the 7 sessions in this experiment consisted of 9 traders who were endowed with “shares” of an asset and amounts of cash, with one treatment endowing subjects with a greater supply of shares (“asset rich”) and another treatment endowing them with a greater supply of lab cash (“cash rich”). Subjects knew that trading would occur in a series of 15 double auctions, after which each share would pay a dividend with a transparently obvious expected value of $3.60. There was no final redemption value for shares, so the fundamental share value was the same, $3.60, irrespective of treatment. As shown in Table 2, all three price averages for the cash-rich treatment shown in the top row are higher than all four price averages for the asset-rich treatment shown in the bottom row.

**Table 2:** Average share prices over all rounds, by Session

Treatment	Mean price per session (all rounds)				Average
Cash rich	3.43	3.73	3.97	–	3.71
Asset rich	3.03	3.32	2.55	3.06	2.99

Caginalp et al. (1998): selected data from Table 2

To investigate how average prices differ between the cash-rich treatment (sample *x*) and the asset-rich treatment (sample *y*), an obvious test statistic is the difference in sample averages:

1 $$T = \overline{x} - \overline{y}$$

The difference, which can be read off the treatment average column in Table 2 as $T_{obs} = 3.71 - 2.99 = 0.72$, is consistent with the “excess cash hypothesis” advanced by the authors. Statistical inference requires comparing this test statistic to its null distribution, however, and—with only three observations in one treatment and four in the other—the usual assumptions required for parametric or asymptotic testing are hard to defend.^3^

At the cost of some statistical power, permutation tests provide a credible basis for statistical inference. Instead of assuming a null distribution for the test statistic, the strategy of permutation testing is to construct this distribution using only the observed data and an understanding of the data generating process.

### Permuting independent measured observations: the Pitman permutation test

Appropriate permutation strategies can be inferred from knowledge of the experimental design and what the null hypothesis would mean for counterfactual sample draws. Here, the null hypothesis of no treatment effect would mean that average prices in every session of the experiment were independent draws from a common average-price distribution. By the same logic, each of the observed price draws would be just as likely to have been assigned to the cash-rich treatment as the asset-rich treatment, so every permutation of the data between these treatments would have an equal ex ante probability of having been observed. With samples of 3 observations in the cash-rich treatment and 4 observations in the asset-rich treatment, there are $\binom{7}{3} = 35$ equally probable ways that these data could have been assigned to the two treatments if the null hypothesis were true. The null distribution of the test statistic can be constructed by computing the value of the test statistic under each of these 35 permutations of the sample data, a subset of which are reproduced in Table 3.

**Table 3:** Computing the null distribution of the test statistic

Permutation index (*i*)	Cash-rich session-average price (*x*)	Asset-rich session-average price (*y*)	Test statistic $T_{i} = \overline{x}_{i} - \overline{y}_{i}$
1	3.03 2.55 3.06	3.43 3.73 3.97 3.32	− 0.73
2	3.03 3.32 2.55	3.43 3.73 3.97 3.06	− 0.58
3	3.32 2.55 3.06	3.43 3.73 3.97 3.03	− 0.56
4	3.43 3.03 2.55	3.73 3.97 3.32 3.06	− 0.52
5	3.43 2.55 3.06	3.73 3.97 3.03 3.32	− 0.50
…	…	…	…
31	3.43 3.97 3.32	3.73 3.03 2.55 3.06	0.48
32	3.73 3.97 3.03	3.43 3.32 2.55 3.06	0.49
33	3.73 3.97 3.06	3.43 3.03 3.32 2.55	0.50
34	3.73 3.97 3.32	3.43 3.03 2.55 3.06	0.66
35*	3.43 3.73 3.97	3.03 3.32 2.55 3.06	0.72

*Observed samples

After permutation values of the test statistic are computed, hypothesis testing becomes straightforward. In conventional hypothesis testing, the *p*-value for a two-sided test represents the probability of drawing a value of the test statistic, *T*, at least as extreme as the observed value, $T_{obs}$, if the null hypothesis were true:

2 $${\text{two-sided}}\;p{\text{-value}} = P_{{H_{0} }} \left( {\left| T \right| \ge \left| {T_{obs} } \right|} \right)$$

This probability is usually calculated by assuming that the test statistic follows a known distribution. Instead of assuming a distribution, permutation testing constructs the empirical null distribution of the test statistic from the sample data. Here, $T_{obs} = 0.72$. There are only two ways that these data could be rearranged to result in a test statistic as extreme or more extreme than $T_{obs}$. These are the permutations in the first and last rows of Table 3. Since the null hypothesis implies that all permutations are equally probable, the *p*-value for a two-sided test of the null hypothesis is $2/35 \approx 0.057.$ More generally, the two-sided *p*-value for this permutation test is the proportion of all test-statistic values greater than or equal to the observed value among all $\binom{m}{n}$ ways of permuting the data between the two samples:

3 $${\text{Pitman}}\;{\text{permutation}}\;{\text{test}},\;{\text{two-sided}}\;p{\text{-value}} = \frac{\sum_{i = 1}^{\binom{m}{n}} 1\left( {\left| {T_{i} } \right| \ge \left| {T_{obs} } \right|} \right)}{\binom{m}{n}}$$

where $T_{i}$ is the value of the test statistic for the *i*th permutation and $1\left( \cdot \right)$ is the indicator function with a value 1 if its argument is logically true and 0 otherwise.

One-sided versions of this permutation test are computed similarly; the only differences are that the numerator consists of signed values of the test statistic and the inequality identifies more extreme observations in the hypothesized direction. A one-sided test for the Caginalp et al. (1998) data could be defended because the experiment was designed to evaluate the excess cash hypothesis that high cash-to-asset ratios would tend to increase share prices. The *p*-value for this one-sided test is the probability of observing a signed value of the test statistic greater than or equal to the observed value under the null. Reviewing Table 3, only the observed permutation meets this criterion, so the one-sided *p*-value is $1/35 \approx 0.029$.

In our experience, students often find the methodology of permutation testing more intuitive than normal-theory testing. Some find it so appealing that they never look back. Even so, it is instructive to consider some of the similarities and differences between the permutation method and the familiar approach of Student’s two-sample *t* test.

First, unlike the *t* or normal distributions, the null distribution of a permutation test can be highly discrete. In the above one-sided example, the *p*-value of 0.029 is the strongest possible rejection of the null hypothesis for these sample sizes. Intuitively, no configuration of the data can be more extreme than the case where all 3 observations in the cash-rich sample are greater than all 4 observations in the asset-rich sample, so the *p*-value for this permutation test could never fall below 1/35 no matter how extreme the difference between the samples.

Second, while having a less discrete null distribution can allow Student’s *t*-test to reach lower *p*-values (0.025 for the two-sided *t* test versus 0.057 in the above two-sided permutation test), it does so at the cost of assuming a specific distribution for the test statistic. This distributional assumption is not innocuous. Inaccurate distributional assumptions can invalidate a parametric test. The permutation test imposes no distributional assumptions in the sense of requiring the sample data to come from a specific population distribution. While the permutation test is not equally powerful for all possible distributions, its validity is not based upon distributional assumptions.

Third, the similarity of this permutation test and Student’s *t* test is not superficial. Both tests are based on permutation-equivalent test statistics (functions of the difference of sample averages). As sample sizes become large, the efficiency of the permutation test converges with that of the *t*-test (Hoeffding, 1952; Miller, 1997: p. 54). Thus, the two-independent-sample permutation test can be seen as a distributionally robust version of the *t* test.

Fourth, while the intuition of permutation testing fits most naturally to hypothesis testing, the methodology can also be used to generate a form of confidence interval (Manly, 2007: pp. 18–20; Miller, 1997: pp. 24, 28). To generate this interval, let $y\left( \theta \right) = y + \theta$ be the observed values of sample $y$, shifted up or down by the value $\theta$. By inspection or numeric search, find the smallest and largest values of $\theta$ such that the permutation test would fail to reject the null of no difference between $x$ and $y\left( \theta \right)$ at level $\alpha$ in a two-sided test. These end points constitute a $100\left( {1 - \alpha } \right)\%$ confidence interval: a range of shift values that could not be rejected at the $\alpha$ level in a two-sided test. Applied to the Caginalp et al. (1998) data, for example, this process yields a 90% confidence interval of (0.244, 1.182). This interval is wider than the (0.266, 1.174) confidence interval produced by the standard *t* test but, unlike the *t* test, does not depend on distributional assumptions.

If everything in this section seems intuitive, one might wonder why this type of permutation testing is not more common in the experimental literature. Indeed, the *Pitman* subcaption of this section is a nod to one of the earliest proponents of this form of permutation testing (Pitman 1937a, 1937b, 1938), yet few experimenters would recognize that name today.^4^ The explanation is that, while the theory of permutation-based inference has been understood for more than 75 years, computing power has only recently made this type of case-by-case construction of null distributions a convenient option (Berry et al., 2019: ch. 2). Recent clearance of computational barriers might raise some doubts about the ease or practicality using permutation tests in published work, but none of them withstand scrutiny.

For example, one might worry that, for large sample sizes, the computational burdens of permutation testing would still become prohibitive even with modern computers. This does happen but it is a problem of little practical importance. For one thing, numerical simulation methods carry the theory of permutation testing past the computational horizon. An approximate permutation test simply replaces exhaustive permutation with many random reshuffles of the observed data.^5^ For another, normal theory and asymptotic testing become defensible as sample sizes grow, meaning the practical need for permutation testing fades just as its computational burdens begin to turn troublesome.

In a similar vein, one might worry that considerable scripting experience would be needed to perform tests that are not already standard routines in common statistical software. Some comfort with scripting is indeed helpful, especially in stratified permutation testing (discussed in Sect. 6) or when designing tests for complicated randomization routines (Heß, 2017). But common statistical programs provide standard routines for permuting sample data; in some cases, available permutation testing libraries make scripting entirely unnecessary. We collect relevant software and routines for permutation testing in online appendixes.

Finally, a pragmatic researcher might hesitate to use an uncommon statistical test for fear that editors and reviewers would question it. We have encountered no evidence of this ourselves. The theory of permutation testing is old and well established. Methods papers have specifically recommended it for experimental work (Moir, 1998). And examples of the use of permutation testing in published work, while not common, are available to cite as precedent if needed. Examples include Sherstyuk (1999), Abbink (2004), Orzen (2008), Anderson et al. (2011), Sieberg et al. (2013), Nosenzo et al. (2015), Rosokha and Younge (2020), Erkal et al. (2020), Kujansuua et al. (2021), Stephenson and Brown (2021), and Schram et al. (2022).

With no remaining obstacles to excuse its unfamiliarity, the Pitman permutation test deserves a more prominent role in the experimenter’s toolkit than it commands today.

### Permuting independent ranked observations: the Mann–Whitney test

While the Pitman permutation test is unfamiliar, related tests devised by Wilcoxon (1945) and Mann and Whitney (1947) are commonly employed in the experimental economics literature. The Wilcoxon and Mann–Whitney approaches describe different but equivalent tests and are sometimes referenced jointly as the Wilcoxon-Mann–Whitney test. For ease of exposition, we refer to both tests as the Mann–Whitney test, which helps to distinguish this permutation strategy from the Wilcoxon Signed Rank test (discussed in relation to matched-pair samples in Sect. 3.2). Just as lack of familiarity with the Pitman permutation test owes to historic and now outdated computational difficulties, the popularity of the Mann–Whitney test owes mainly to inertia from computational shortcuts of little modern importance.

The Mann–Whitney procedure presented in most non-parametric books involves ranking all sample data (both samples combined) and replacing each observation with its ordinal rank in the combined sample. Both the Wilcoxon and Mann–Whitney versions of the test then compute special test statistics with computationally convenient null distributions (Gibbons & Chakraborti, 2003; Miller, 1997; Siegel, 1956). The specific definition of the test statistic is not important for present purposes. The thing to note is that the approach is equivalent to running a permutation test on the rank-transformed sample data.

Specifically, the Mann–Whitney test is a two-independent-sample permutation test (covered in Sect. 2.1) in which the data being permuted are not the measured observations but the ordinal ranks of those observations in the combined sample (Siegel, 1956: p. 155). Instead of permuting observed average prices in the Caginalp et al. (1998) experiment, the Mann–Whitney test would permute the ranked values of each session-average price in the combined sample. The cash-rich average prices of 3.43, 3.73, and 3.97 were the three highest prices observed in this experiment, with ranks 5, 6, and 7 respectively; the asset-rich average prices of 3.03, 3.32, 2.55, and 3.06 were the four lowest prices, with ranks of 2, 4, 1, and 3 respectively. The Mann–Whitney test is equivalent to running a Pitman permutation test on these rank-transformed observations.

It happens to make no difference, in this example, whether one works in ranks or level data. Since the ranked versions of the samples, *x* = {5, 6, 7} and *y* = {2, 4, 1, 3}, are more extreme than any other possible permutation, the one-sided *p*-value is still $1/35 \approx 0.029$ and the two-sided *p*-value is still $2/35 \approx 0.057$. This is a special case, however, and permutation tests based on ranks are not generally the same as those based on observed values.

To illustrate the potential difference between these tests, consider an experiment reported by Bohr et al. (2019), involving asset market performance with saving decisions over a simulated lifetime. Subjects, in this experiment, were permitted to buy and sell asset “shares” that paid dividends each period. The dividends and interest paid on cash induced a flat fundamental share value (present value of future dividends) of $20 per share. In six sessions of the experiment (the “private-savings” treatment), subjects traded assets while also deciding how much of their incomes to save for low-income “retirement”﻿ years. In another 6 sessions (the “government-savings” treatment), a fixed portion of each subject’s income was withheld by the “government” for retirement years. As a result of the difference in savings policy, subjects in the private-savings treatment carried cash amounts that were about twice as large as those in the government-savings treatment. Peak price data are provided for both treatments in Table 4, below.

**Table 4:** Peak price data and ranks for asset shares

Treatment	Session peak prices	Mean					
Private savings	42	36	53	61.5	38.5	70	50.2
Government savings	42.5	21.25	30	26	43	38	33.5

Bohr et al. (2019): selected data from Table 1

While major price bubbles (above the $20 fundamental value) were observed in most sessions of the experiment, an interesting research question is whether peak asset prices were greater under the cash-rich private-savings treatment than under the government-savings treatment.^6^ The data are qualitatively consistent with this hypothesis but there is overlap between the samples. When permuting the measured observations under the Pitman test, there are $$\binom{12}{6} = 924$$ possible permutations of the 12 observations across treatments, 36 of which yield a treatment difference at least as extreme as the observed value, for a two-sided *p*-value of about 0.039. In contrast, when permuting rank-transformed observations under the Mann–Whitney test, there are 86 permutations in which the treatment difference (in average ranks) is at least as extreme as the observed value, yielding a two-sided *p*-value of about 0.093. The rank-based Mann–Whitney test barely supports rejection of the null hypothesis at the 10% level, while the Pitman test’s greater use of the sample information allows for rejection at the 5% level.^7^

Since the Pitman permutation test is sensitive to the magnitudes of observation differences, not just their rank comparisons, one might think that the *p*-values produced by Pitman tests will always be lower than those of equivalent Mann–Whitney tests. This intuition is incorrect. To illustrate, consider an experiment reported by Holt and Smith (2022) in which subjects competed for a fixed monetary prize by expending lab dollars on “effort” that has a specified cost per unit. One treatment contrast compared average total expenditures on “rent-seeking” activity with low effort cost (77, 83, 130, 81) against expenditures with high effort cost (132, 111, 93, 87).^8^ Of the $\binom{8}{4} = 70$ possible ways of permutating the sample observations, 28 yield a treatment difference more extreme than what was observed, making the *p*-value of a two-sided Pitman test $28/70 = 0.4$. After rank-transforming the sample observations, however, only 14 of 70 permutations yield an average difference more extreme than the observed value, meaning the *p*-value of a two-sided Mann–Whitney test is $14/70 = 0.2$.

Another way that the Pitman and Mann–Whitney tests often differ is in confidence intervals. Recall that the Pitman test produced a 90% confidence interval of (0.244, 1.182) when assessing the difference between cash-rich and asset-rich average prices in the Caginalp et al. (1998) experiment. Confidence intervals can also be generated for rank-transformed tests (Bauer, 1972). Computing the end points of an interval by searching for the largest and smallest shift-terms where a two-sided Mann–Whitney test would not reject the null hypothesis at the 90% level yields a 90% confidence interval of $\left( {0.111, 1.420} \right)$.

If the only difference between the Mann–Whitney and Pitman permutation tests is that the Mann–Whitney tests drops information when sample data are measured as interval values, then why is the Mann–Whitney test so popular? It once enjoyed the important advantage of having a null distribution that could be pre-computed and printed in critical-value tables in statistics textbooks. But modern computing power makes this all but irrelevant. The rank-based test is still appropriate when the sample data are ordinal as measured.^9^ And, because the rank conversion suppresses distortions caused by outliers, the Mann–Whitney test may also be preferable to the Pitman test when experimenters are concerned that outliers might be introduced by measurement errors, procedural issues, or other artificial influences.

In most cases involving interval measurements, however, the Pitman test presents the more compelling option. The Pitman test uses more of the sample information and is more sensitive to differences between measured observations. In a Monte Carlo comparison of the Pitman test against Student’s *t*-test and the Mann–Whitney test, Moir (1998) finds the Pitman test to match or outperform the more familiar tests in most applications.^10^ Where data are interval as measured (prices, auction revenues, market efficiencies), and where policy interest concerns the magnitudes of treatment effects, it is hard to justify passing over the Pitman test in favor of an alternative that is less sensitive to these aspects of the data.

## Permutation tests for *k* = 2 matched pairs

An important distinction when analyzing experimental data concerns the difference between *within-subjects* designs and *between-subjects* designs. In between-subjects designs, each person or group is exposed to a single experimental treatment. This produces samples of independent observations (the focus of the previous section). Within-subjects designs expose each person or group to multiple treatments in sequence. Exposure to more than one treatment has the potential drawback that behavior induced by one treatment may carry over to another treatment—a design bias known as an *order effect*. But within-subjects designs have the advantage of collecting data in a way that allows each person or group to serve as its own control group—a potentially valuable property when considering treatment effects in the presence of heterogeneity.

In the two-treatment context, within-subjects designs produce samples consisting of matched pairs of observations. For example, if measurements are taken when $n$ subjects are exposed to a control condition of an experiment, $\left( {x_{1} , \ldots ,x_{n} } \right) \sim F_{x}$, and measurements are repeated when the same $n$ subjects are exposed to a treatment condition of the experiment, $\left( {y_{1} , \ldots ,y_{n} } \right) \sim F_{y}$, then the difference vector $\left( {d_{1} = x_{1} - y_{1} , \ldots ,d_{n} = x_{n} - y_{n} } \right) \sim F_{x - y}$ reflects how exposure to the treatment has changed the measured outcome *within* each subject in the experiment. It is convenient to work directly from this difference vector when testing hypotheses using this type of matched-pair data. The null hypothesis of no treatment effect, $F_{x} = F_{y}$, corresponds to a difference distribution, $F_{x - y}$, that is symmetric about 0, such that differences between the control and treatment are explained by random noise alone. The alternative hypothesis of treatment distributions differing in central tendency corresponds to a difference distribution with non-zero central value—again, assuming a shift model.

A helpful illustration of a within-subjects design is an experiment created to study how prices respond to changes in the number of sellers and market power in a posted-price oligopoly. Davis and Holt (1994) assigned each of 12 sessions of an experiment to two of three treatments. Six of the sessions entailed 30 periods of price competition followed by 30 periods of competition under a redistribution of production capacity that created or reduced market power, holding the number of sellers fixed.^11^ The other six sessions entailed 30 periods of price competition followed by 30 periods of competition with the addition or removal of 2 sellers from the market, holding market power fixed.^12^

Table 5 shows observed price measures for this experiment. The numbers in the table are average prices over the final 15 replications of a market treatment—that is, the later replications in which strategies and behavior have had time to reach steady states. Asterisks on session labels denote those sessions in which subjects were first exposed to the topmost of the two treatments. Orthogonal treatment assignment was intended to mitigate the potential design bias caused by any order effects in the experiment.

**Table 5:** Average prices for different oligopoly markets

Treatment/session	S1*	S2	S3*	S4	S5*	S6	S7*	S8	S9*	S10	S11*	S12
5 sellers no market power	329	308	341	410	310	397						
5 sellers market power	407	468	430	455	397	441	415	471	392	401	392	512
3 sellers market power							425	470	408	436	424	517

Davis and Holt (1994): Table 1, page 479

*The top treatment was the first exposure in this session

The researchers sought to answer two related questions. First, does the market power created by redistribution of production capacity to larger sellers confer pricing power? Second, even holding market power constant, is there a pure *numbers effect* in which fewer competitors means greater pricing power? In both cases, the data appear to reflect a treatment effect, but with only 6 matched-pair data points for each comparison, tests based on assumed distributions are again hard to defend. A permutation approach is more persuasive.^13^

### Permuting pairs of measured observations: the Fisher permutation test

As before, the appropriate permutation strategy for constructing the null distribution of a test statistic can be inferred from knowledge of the experimental design and what the null hypothesis would mean for counterfactual sample draws. Here, the null hypothesis of no treatment effect implies that the vector-difference of matched pairs should have zero mean, suggesting the use of the average difference as an appropriate test statistic:^14^

4 $$T = \overline{d}$$

As noted above, the null hypothesis implies that the difference distribution should be symmetric around zero. This motivates a simple permutation strategy for constructing the null distribution of the test statistic.

Since every difference is equally likely to be either positively or negatively signed under the null hypothesis, the null distribution of the test statistic can be computed by recalculating the value of the test statistic under all possible permutations of the signs of the sample differences. Equivalently, and perhaps more intuitively, since $F_{x}$ and $F_{y}$ are equal under the null hypothesis, the observed values in every matched pair are equally likely to have been assigned to opposite treatments under the null—which corresponds to simply swapping the sign of their difference. This strategy of permuting signs is often attributed to Fisher (1935). We refer to it as *the Fisher permutation test.*

For a sample of $n$ matched pairs, there are $2^{n}$ ways that the signs of all matched pairs could be permuted under the null. Taking the *p*-value to be the probability of seeing a value of the test statistic, *T*, as or more extreme than the observed value, $T_{obs}$, a permutation *p*-value is as follows:

5 $${\text{Fisher}}\;{\text{permutation}}\;{\text{test}},\;{\text{two-sided}}\;p{\text{-value}} = \frac{{\mathop \sum \nolimits_{i = 1}^{{2^{n} }} 1\left( {\left| {T_{i} } \right| \ge \left| {T_{obs} } \right|} \right)}}{{2^{n} }}$$

where $1\left( \cdot \right)$ is again the indicator function.

The Davis and Holt experiment provides a concrete context for illustration. Recall that the first question is whether increased market power confers pricing power in this setting. For the 6 matched pairs in sessions S1–S6 of Table 5, the average price is higher for the market power treatment in every case. That is, the sample-difference vector has only positive signs. Since there are $2^{6} = 64$ possible permutations of the signs of these matched pairs under the null, only two of which result in a test statistic as or more extreme than the observed test statistic, the two-sided *p-*value is $2/64 \approx 0.031$. Since economic theory predicts a positive sign for this treatment effect, it is arguably more appropriate to use a one-sided test in this setting. The one-sided *p*-value corresponding to a test of the alternative hypothesis that prices are higher with greater market power seeks only those permutations with test statistic values greater than or equal to the observed value. Since no alternative permutation of the signs of these data provides an average value larger than what was observed, the one-sided *p*-value is about $0.016$. This example is, however, misleading in its simplicity. Since all differences were of the same sign, the magnitudes of differences did not need to be considered in calculating the $1/64$ probability for each tail. Next, we consider a more realistic example with some overlap.

The second question is whether a pure *numbers effect* gives smaller numbers of competitors greater pricing power, even when holding technological market power constant. For the 6 matched pairs in sessions S7–S12 of Table 5, the average price is greater under the 3-seller treatment in all but one case. Focusing on the one-sided test, 2 possible permutations yield an average difference greater than or equal to the observed value. The *p*-value of a one-sided test is thus $2/64 \approx 0.031$; the corresponding *p*-value for a two-sided test would be $4/64 \approx 0.062$.

Note that the previous conclusion would not have been reached if testing had not accounted for matched pairs in this design. If the 3-seller power and 5-seller power samples on the right side of Table 5 had been treated as independent samples, a Pitman permutation test would give a one-sided *p*-value of about 0.273, which would not support rejection of the null hypothesis of no numbers effect. The intuitive explanation for this is that groups of subjects can differ in competitiveness. The most collusive outcome observed in any 3-seller market was produced by the same group that produced the most collusive outcome in any 5-seller market. Using each group as its own control helps to mitigate the effects of subject heterogeneity and thus helps to reveal treatment effects that might otherwise be difficult to distinguish from noise in the data.

Like the Pitman test, the Fisher permutation test has appeared in published articles. Examples include Baillon, Schlesinger, and van de Kuilen (2018) and Bao et al. (2020).

### Permuting ranked observations: the Wilcoxon signed-rank test

Just as the ability to perform permutation tests on measured data is often overlooked in experimental analysis of independent samples, so is it overlooked in the matched-pairs context. By far, the most common small-sample test in the matched-pairs setting is the Wilcoxon (1945) signed-rank test. The name of this test reflects the peculiar transformation that it applies to the measured data. All matched-pair differences are ranked from smallest to largest in absolute value, and these ranks are then reassigned the signs of the original difference data. To illustrate, the vector of sample treatment differences $d = \left( { - 6, 4, 0, - 3} \right)$ becomes $SR_{d} = \left( { - 4, 3,1, - 2} \right)$.

As was the case for the Mann–Whitney test, the original motivation for using the signed-rank transformation was primarily to enable reliance on a test statistic for which a precompiled null distribution could be provided in printed form. The specifics of the relevant test statistic and its distribution are interesting but are not belabored here. Accessible presentations are provided by Wilcoxon (1945) and most introductory texts on nonparametric statistics (Gibbons & Chakraborti, 2003; Miller, 1997; Siegel, 1956). It is sufficient to note that the Wilcoxon signed-rank test is a Fisher permutation test conducted on the signed ranks of the sample difference vector.

How do the Fisher permutation test and the Wilcoxon signed-rank test compare? For the Davis and Holt experiment, both yield the same *p*-values. Fisher’s permutation test has strong intuitive appeal as the permutation analog of Student’s one-sample *t*-test (Miller, 1997). Monte Carlo evidence demonstrates the superior power of the Fisher permutation test over the Wilcoxon signed rank test for a variety of sample sizes and distributions (Kempthorne & Doerfler, 1969). This is intuitive, as a Fisher permutation test run on interval data uses more sample information than does a Wilcoxon signed-rank test. In our opinion, the Fisher permutation test should be the experimenter’s default choice when differences between observed measurements reflect important outcomes—with the Wilcoxon test reserved for situations in which data are ordinal as measured or when outliers are a concern.

To illustrate the last point, suppose that the first session of the 5 seller no-market-power treatment had yielded an observed price of 700, rather than 329. Since the Fisher permutation test is based on measured values instead of ranks, the magnitude of this observation affects the test statistic quite a bit. When considering the one-sided alternative, there are now 26 possible sign permutations that yield average differences greater than or equal to the observed difference, for a *p*-value of $26/64 \approx 0.406$. The magnitude of the high-price observation has less effect when converted to a signed rank in the Wilcoxon test. When considering the one-sided alternative, there are only 14 possible sign permutations that would yield an average difference of signed ranks greater than or equal to the observed value, for a *p*-value of $14/64 \approx 0.219$. The Fisher permutation test’s sensitivity to extreme observations is a strength of the test when these observations reflect underlying economic conditions but is a weakness when these observations are artificial outliers.

## Permutation tests for *k* > 2 independent and unordered samples

The permutation tests discussed thus far have involved comparisons of two treatments, but many experiments involve more than two treatments. While multiple pairwise comparisons can always be conducted in this setting, there are downsides to this approach. For one thing, it can be difficult to interpret situations in which some tests justify rejection of the null hypothesis while others do not. True, these differences in outcome may be important findings in some cases, but in other cases they can frustrate efforts to state succinctly whether a relationship was strongly rejected by the data. For another thing, the simultaneous assessment of multiple tests may require multiple-comparison adjustments to control for error inflation, leading to power loss.

Simultaneous tests designed to detect locational differences among a set of more than two samples can be an attractive alternative. Two common examples of this approach are tests of multiple unordered treatment effects and tests of treatment effects with ordered predicted intensity. This part will take up the case of unordered treatment effects, first with measured data and then with rank-transformed data.

### Permuting measured observations: the *F* test

The test of multiple unordered treatment effects is a simple generalization of the test of locational difference between two independent samples. In the two-sample setting, samples $x$ and $y$ are compared to see whether their means are statistically distinguishable. In the more general setting, $k > 2$ samples, $x_{1} , \ldots ,x_{k} ,$ of size $n_{1} , \ldots ,n_{k}$ are compared simultaneously to see whether any of their means are statistically distinguishable. If experiment sessions are run at *k* different universities, for example, a researcher might want to determine whether any subject pool differences are revealed. In statistics textbooks, this procedure falls under the heading of one-factor analysis of variance (ANOVA) testing, and statistical inference would most likely be based on the *F* statistic and its comparison to the *F* distribution:

6 $$F = \frac{{\left( {k - 1} \right)^{ - 1} \mathop \sum \nolimits_{j = 1}^{k} n_{j} \left( {\overline{x}_{j} - \overline{x}} \right)^{2} }}{{\left( {N - k} \right)^{ - 1} \mathop \sum \nolimits_{j = 1}^{k} \mathop \sum \nolimits_{i = 1}^{{n_{j} }} \left( {x_{i,j} - \overline{x}_{j} } \right)^{2} }}$$

where $x_{i,j}$ denotes the *i*th observation in sample $x_{j}$, $\overline{x}_{j}$ and $n_{j}$ denote the mean and number of observations in sample $x_{j}$, $\overline{x}$ denotes the grand mean when all samples are pooled together, and *N* is the total number of observations. This test is known to be robust against non-normality (Miller, 1997: 80–81), but experimental data may push the boundaries of what could plausibly justify the assumption of a specific, parametric null distribution.

Applications of permutation testing in one-way ANOVA go back nearly as far as the use of permutation testing in the two-sample setting (Pitman, 1938). The reason is that the two-sample permutation process generalizes in an intuitive way to the higher order setting. To illustrate, consider an experiment that Holt and Sprott (2022) use to evaluate different methods for auctioning off-shore wind energy leases. One treatment implements a clock auction used by the US Department of Interior. The initial clock price is set low to generate excess demand for leases, and the clock price is raised in a sequence of rounds until there is no excess demand, which determines the uniform sale price. A second treatment models the multi-round sealed-bid procedure used in the UK. In that auction, the high bidder in the first round receives any leases they bid for at their own bid price, and these leases are removed from the auction. The high bidder in the second round then gets any leases they bid for at their bid price, and so on until all leases are sold.^15^ Finally, a third treatment implements a single-round sealed-bid auction. After the bids are submitted, the auction is cleared by going down the bid list from high to low and accepting bids until no leases remain unsold. Winning bidders pay their own bid amounts.

Table 6 shows average earnings over each of 4 sessions of each treatment. Since the costs of wind-energy generation are difficult to estimate precisely in advance, the experiment was structured so that each bidder received independent noisy signals of the underlying lease value. This common-value setting entails a risk that the winning bidder might have bid high because they overestimated the lease value. This well-known “winner’s curse” effect is clearly indicated by the negative average earnings for the UK auction treatment in the top row.

**Table 6:** Wind energy auction earnings

Treatment	Session-average earnings				Average
UK (right to choose auctions)	− 0.61	− 3.64	0.82	1.39	− 0.51
US (clock auctions)	3.84	1.04	3.17	3.97	3.00
Single-round pay-as-bid auction	0.22	− 0.24	0.42	1.13	0.38

Holt & Sprott (2022): data on file with authors

To illustrate how a permutation test could be applied to these data, consider a test of the null hypothesis of no difference between any of the auction formats, against the alternative of a locational difference between at least two of the mechanisms. The observed data yield an *F* statistic of 5.54. All that remains is to construct the null distribution and to see how the observed value of the test statistic compares with it.

As before, the null distribution can be derived from knowledge of the experimental design and the implications of the null hypothesis. If the null of no treatment effect were true, then all observations in the experiment would be equally likely to have been assigned to any of the treatments. If there are $k > 2$ treatments with $n = \sum n_{k}$ total observations, then there are $\binom{n}{n_1}$ ways that observations could have been assigned to the first treatment. For each of these, there are $\binom{n-n_1}{n_2}$ ways that observations could have been assigned to the second treatment. For each of these, there are $\binom{n - n_1 - n_2}{n_3}$ ways that observations could have been assigned to the third treatment. And so on, for a total of $n!/\left( {n_{1} ! \times \ldots \times n_{k} !} \right)$ equally likely permutations of the sample data under the null. The *p*-value for a permutation-based test of the null hypothesis (equality of all samples) comes from computing the *F* statistic for every permutation of the data and counting the proportion of these *F* statistics that are greater than or equal to the observed value:

7 $${\text{permutation}}\;F\;{\text{test}},\;p{\text{-value}} = \frac{{\mathop \sum \nolimits_{i = 1}^{N} 1\left( {F_{i} \ge F_{obs} } \right)}}{N}$$

where $N = n!/\left( {n_{1} !\; \times \; \ldots \; \times \;n_{k} !} \right)$. For the wind auction experiment, three groups of four observations yields $N = 12!/\left( {4!\; \times \;4!\; \times \;4!} \right) = 34,650$ possible permutations of the observed data. As noted above, the observed value of the test statistic is 5.54. Of the 34,650 possible permutations of the sample data, 642 yield *F* statistics greater than or equal to 5.54, so the *p*-value for a permutation *F* test of the null hypothesis is about 0.019. This *p*-value is smaller than the *p*-value of $4/70 \approx 0.057$ that comes from pairwise comparison of even the highly contrasting US and UK treatments using the Pitman test discussed in Sect. 2.1. Of course, specific pairwise comparisons would be relevant for the actual choice of an auction format.

### Permuting ranked observations: the Kruskal–Wallis test

The above description of the permutation *F* test differs from the familiar Kruskal–Wallis test—the current default choice of most experimenters when seeking a nonparametric test of locational difference among *k* samples (Kruskal & Wallis, 1952). Like the Wilcoxon and Mann–Whitney tests, the Kruskal–Wallis test statistic is a function of rank-transformed observations:

8 $$H = \left( {N - 1} \right)\frac{{\mathop \sum \nolimits_{j = 1}^{k} n_{j} \left( {\overline{r}_{j} - \overline{r}} \right)^{2} }}{{\mathop \sum \nolimits_{j = 1}^{k} \mathop \sum \nolimits_{i = 1}^{{n_{j} }} \left( {r_{i,j} - \overline{r}_{j} } \right)^{2} }}$$

where $r_{i,j}$ denotes the rank (among all observed values) of the *i*th observation in sample $x_{j}$, $\overline{r}_{j}$ and $n_{j}$ denote the mean rank and number of observations in sample $x_{j}$, $\overline{r}$ denotes the average of all ranks, and *N* is the total number of observations.

How does the Kruskal–Wallis Test compare to the permutation *F* test? Consistent with our discussion in the two-sample context, rank-based tests make sense when the sample data are ordinal as measured, or when the experimenter has reason to be concerned about serious outliers not connected to fundamentals of the subject being investigated. There is, however, an important difference between the Kruskal–Wallis test and the previously discussed two-sample rank-based tests. Unlike its two-sample analogs, the Kruskal–Wallis test does not have an easily computed null distribution. Implementations in common statistics software substitute approximate null distributions*,* but these are known to be poor approximations for small sample sizes (Meyer & Seaman, 2013). The exact null distribution can be constructed by permutation, but then the Kruskal–Wallis test lacks any computational advantage over the permutation *F* test. For these reasons, rote reliance on the Kruskal–Wallis test is not advisable.

To illustrate how these considerations play out in an actual example, return to the Wind Auction experiment and the null hypothesis of no difference in treatment effect between any of the auction mechanisms. Recall that the permutation *F* test rejected the null hypothesis with a *p*-value of 0.019. An exact *p*-value for the Kruskal–Wallis test can be computed by following the same process, only replacing the *F* statistic with the *H* statistic. For the Wind Auction data, the observed value of the test statistic is $H = 5.69$. Of the 34,650 possible permutations of the sample data, 1,686 yield *H* statistics greater than or equal to 5.69, making the exact *p*-value of the rank-based Kruskal–Wallis test $1,686/34,650 \approx 0.049$.

## Permutation tests for *k* > 2 independent and ordered samples

In the previous discussion, the direction of the alternative hypothesis was left unspecified; the null hypothesis of no treatment effect was compared to the agnostic alterative that at least two of the treatments differed from each other in central tendency. This alternative will often be appropriate, but sometimes experiments are designed so that treatments differ in intensity along a single dimension: for example, group size or the incentive to defect from a cooperative outcome in a social dilemma. In these cases, a directional alternative hypothesis may be appropriate.

While something like the all-purpose permutation *F* test is sensitive to the presence of ordered treatment effects—and thus a valid test even when an ordered treatment effect is expected—more powerful tests may be constructed to test for ordered effects. Much like the difference between one-sided and two-sided tests in the two-treatment context, tests of ordered treatment effects are preferable when theory suggests this hypothesis.

Where the relationship between treatments and hypothesized effects is approximately linear, correlation and regression methods provide an attractive basis for inference. We discuss the use of permutation methods in these models of association in Sect. 7. Here, we consider situations where linearity cannot be assumed. Examples include situations where treatments differ by broad, qualitative distinctions (such as when subjects are categorized into bins like risk averse, risk neutral, or risk seeking) or where experimenters expect to see ordered treatment effects but cannot predict more than the ordinal sequence of the relationship.

### Permuting ranked observations: the Jonckheere–Terpstra test

To ground discussion of ordered alternative hypotheses, consider the classic Smith (1964) experiment comparing three variations of the double-auction trading institution. Based on observations of a prior pilot experiment, Smith conjectured that prices would be lower when sellers competed against and undercut each other and that prices would be higher when buyers bid against each other to compete for purchases. To test this, Smith ran two sessions for each of three treatments—each session lasting 5 rounds—with infra-marginal buyer values and seller costs creating identical supply–demand arrays symmetrically configured around a unique equilibrium price in every round. In a sellers-offer treatment, sellers could make price offers during a trading period, whereas buyers could only observe offers and decide whether to accept them. The situation was reversed in a buyers-bid treatment: only buyers could make bids to purchase, and sellers were constrained to observe and decide whether to accept bids. Finally, the double-auction treatment is symmetric: buyers could make bids, sellers could make offers, and either side could accept a bid or offer. Sessions in groups A and B were run with 20 and 24 traders, respectively. Here, we rely on the theoretical invariance of equilibrium outcomes to group size to pool the two session-average prices in each column of Table 7 (yielding 2 observations per treatment), but we will return to this group-size nuisance variable in Sect. 6.2.^16^ Table 7 shows average prices for the final two trading periods in each session. The null hypothesis is that the trading rules have no effect on price levels. The alternative hypothesis is that prices will be lowest in the sellers-offer treatment and highest in the buyers-bid treatment.

**Table 7:** Session-average contract price by trading condition

Group	Sellers offer	Double auction	Buyers bid
Group A (20 traders)	208	213	217
Group B (24 traders)	195	209	213

Smith (1964): selected data from Table 3

The most commonly used nonparametric test when testing for this type of ordered treatment effects is the Jonckheere–Terpstra test (Jonckheere, 1954; Terpstra, 1952. When treatment categories are ordered so that the predicted effect increases from left to right (as in Table 7), the test statistic *J* is the sum of all “binary wins” in the predicted direction. In other words, *J* is the total number of larger observations in columns to the right of each observation:

9 $$J = \mathop \sum \limits_{s = 1}^{k - 1} \mathop \sum \limits_{t = s + 1}^{k} \mathop \sum \limits_{i = 1}^{{n_{s} }} \mathop \sum \limits_{j = 1}^{{n_{t} }} 1\left( {x_{i,s} < x_{j,t} } \right)$$

where $x_{i,s}$ is the *i*th observation in the sample data from ordered treatment *s*, $x_{j,t}$ is the *j*th observation in the sample data from ordered treatment $t > s$, $1\left( \cdot \right)$ is the indicator function, and $n_{s}$, and $n_{t}$ are respective sample sizes. Notice that the first summation, indexed by *s*, is over all columns except the final column, *k*. The second summation, indexed by *t*, is over all columns to the right of *s*, up to and including column *k*. The final two summations are over all pairs of observations in columns *s* and *t*, which are used to obtain a “less-than” count via the indicator function. Here, the average contract price of 208 for the Group B session in the Seller Offers treatment is smaller than all 4 numbers in columns to its right, so the first term in the sum for *J* would be a 4. Similarly, the contract price of 195 is smaller than all 4 numbers to its right, so the second term in the sum for *J* is also 4. In the middle column, the contract price of 209 is smaller than 2 numbers to its right, so the third term in the sum is 2, but the contract price of 213 in the middle column is only smaller than 1 number to its right, so the final term in the sum is 1. The sum of these 4 terms yields the test statistic, $J_{obs} = 4 + 4 + 2 + 1 = 11$.

For large sample sizes, approximate null distributions for *J* are available. For small samples like this, the researcher is left to search for a precomputed null distribution or to generate one via permutation. By the logic used in Sect. 4.1, the null hypothesis of no treatment effect implies that there are $n!/\left( {n_{1} !\; \times \; \ldots \; \times \;n_{k} !} \right)$ equally likely permutations of the observed sample data. The permutation *p*-value for this test is thus computed the same way as the *F* test described in Eq. (7) but with $J_{i}$ and $J_{obs}$ substituted in place of $F_{i}$ and $F_{obs}$. Here, the observed value of the test statistic is $J_{obs} = 11$ and, of the $N = 6!/\left( {2!} \right)^{3} = 90$ possible permutations of the data, only 2 yield a value of *J* as or more extreme than the observed value of 11, so the *p*-value for the Jonckheere–Terpstra test is $2/90 \approx 0.022$.

The Jonckheere–Terpstra test is an attractive option when sample data are measured as ordinal values. It is also a test that appears with some regularity in the literature. Smith (1964) used a Jonckheere–Terpstra test in his analysis of these data.^17^ Other recent examples include Gürerk and Selten (2012) and Conrads et al. (2016). When sample data are interval as measured, however, the Jonckheere–Terpstra test operates like other rank-based tests in discarding sample information. The obvious question is whether a different test could be constructed that does not involve this loss of information.

### Permuting measured observations: a directional difference test

How might a magnitude-sensitive version of the Jonckheere–Terpstra test be constructed? A simple approach would be to replace the “binary win” count with a sum of differences.^18^ With the treatment vectors of observations still listed left to right in increasing order of predicted effect, let the test statistic *D* be the sum of all differences between each observation and all observations in columns to the left in the ordered array:

10 $$D = \mathop \sum \limits_{s = 1}^{k - 1} \mathop \sum \limits_{t = s + 1}^{k} \mathop \sum \limits_{i = 1}^{{n_{s} }} \mathop \sum \limits_{j = 1}^{{n_{t} }} \left( {x_{j,t} - x_{i,s} } \right)$$

where all terms are defined as above. For the Smith (1964) data, the observed test statistic is $D = 108$, which is equaled or exceeded by only 2 test statistics in the 90 possible permutations of the data, for a *p*-value of $2/90 \approx 0.022$. Here, the Jonckheere–Terpstra test (based on binary win comparisons) and the Direction Difference test [based on calculated directional differences in (10)] yield the same *p*-values. This is a special case, however, and these tests will not generally yield the same results when applied to the same data. Rather than belabor this difference, which we have already seen in other applications, we note two points.

First, as in other comparisons, the availability of a test that operates on measured observations demands an explanation for preferring a rank-based test when sample data are interval as measured. Unless a precomputed null distribution is available, the computational cost of calculating exact *p*-values for the Jonckheere–Terpstra test is the same as for the Directional Difference test. We conjecture that the Jonckheere–Terpstra test could have superior properties when the samples contain outliers, though we are aware of no Monte Carlo analysis or proof to this effect. Absent special considerations, the Directional Difference test provides a more natural and intuitive basis for statistical inference.

Second, there is potentially great research value in designing experiments with the type of treatment variation illustrated in this example. Suppose that, instead of collecting two data points from each of three treatments, the researchers had followed the more conservative approach of collecting three data points for each of two treatments. The strongest possible rejection by a permutation test in the two-treatment context would be at a *p*-value of $1/20 = 0.05$ for a one-tailed test, assuming no reversals between the treatments. In Table 7, with the same number of data points spread across three treatments, the *p*-value is smaller even in the presence of a tied “reversal” for the 213 price in each of the double-auction and buyers-bid treatments.

Several generalizations of the Jonckheere–Terpstra test have been used for physical systems in which too much of a treatment may have a negative effect. The “umbrella test” is for the case where the alternative hypothesis involves a “hill-shaped” data pattern as treatment intensity is increased (Mack & Wolfe, 1981). This test essentially combines two directional tests: one for data to the left of the mode, and one for data to the right. Analogous test statistics could be devised for the directional difference test. This illustrates an intriguing property of permutation testing. As Pearson (1937) observed at the dawn of this methodology, the permutation method’s decoupling of computation of the null distribution from choice of test statistic frees researchers to select whatever test statistic is most sensitive to a hypothesized relationship.

## Stratification and permutation tests for *k* > 2 dependent samples

Just like the independent-sample permutation procedures, matched-sample procedures generalize in an intuitive way to higher order settings with *k* treatments per observational unit. In classical statistics texts, the study of treatment effects when the same subjects are exposed to multiple treatments in sequence is presented under the heading of two-way analysis of variance. In the permutation context, we find it easier to conceptualize the data generating process in terms of stratification. The following illustrates how stratified permutation approaches address common data challenges, first in a two-treatment context with discrete nuisance variables and then in the general case of multiple sample comparisons.

### Stratified permutation tests for *k* = 2 treatments with nuisance variables

A common problem in experimental data analysis is dealing with procedural differences in data groupings that are unrelated to the difference of interest. These procedural groupings are essentially nuisance variables: secondary treatments that ideally would be held constant when evaluating the effects of the primary treatments. For example, suppose an experiment involves two treatments, each applied to subjects from two different pools. If the experimenter has reason to believe that these subject pools are interchangeable, then observations can be pooled by treatment and a simple two-sample test can be employed. If subject pools cannot be assumed to be interchangeable, however, then the need to perform separate tests for each subject pool can present both narrative and statistical challenges (a point we illustrate below).

Stratified permutation testing may be a more attractive option. To explain what we mean, let the primary treatments be indexed by *j*, and let the secondary groupings be indexed by *g*. Thus, $x_{ijg}$ denotes experimental observation *i* taken when treatment *j* is applied to subjects from group *g*. The idea behind stratified permutation testing is to construct the null sampling distribution by permuting the primary treatment labels (*j*) *within* groups but not *between* groups. This procedure captures the null hypothesis—that observed measurements are equally likely to be seen under any treatment—without imposing the additional assumption that observed measurements are equally likely to be seen under any secondary grouping.

As a concrete example, consider the two-treatment asset market experiment reported by Holt et al. (2017). One treatment, applied to 14 sessions, involved a 25-period trading sequence; the other treatment, applied to 10 sessions, involved a 15-period trading sequence. These markets were blocked on gender: half of the sessions in each treatment were female-only and half were male-only. In all treatments, the fundamental (present) value of asset shares was constant, at $28, for all trading periods. Price bubbles, with peaks well above $28, were observed in all sessions. Table 8 shows peak asset prices for female-only sessions (top row) and male-only sessions (bottom row). Shorter sessions afforded subjects less opportunity to accumulate large cash balances, resulting in smaller cash-asset-value ratios. This difference in cash-asset values motivated a question whether the peak asset prices were also lower in the shorter markets. For purposes of testing the null hypothesis of no trading-length effect, trading-length is the control variable of primary interest and gender groupings are a nuisance variable.^19^

**Table 8:** Peak prices by market with gender sorting

Market pool	25-period markets	15-period markets	Mean
Female only	87 95 61 177.5 75.5 37 152	66 36 58 64 42	79.3
Male only	55 48 68 85 65 56.5 50	50 70 45 43 53	57.4
Mean	79.5	52.7	68.3

Holt et al. (2017): selected data from Tables 1 and 5

In order to evaluate the effect of the number market periods, while controlling for gender, a stratified permutation test permutes peak price observations across session-length treatments (the columns of Table 8), but not across gender-groups (the rows of Table 8). A one-sided permutation test of the null of no treatment effect against the alternative of higher price peaks in longer markets can be based on the observed difference in the bottom row of the table, $T_{obs} = 79.5 - 52.7 = 26.8.$ The null distribution of this test statistic is constructed by computing the test statistic over each of the constrained set of permutations in which observations are moved between market-length treatments but not across gender labels. There are $\binom{12}{5} = 792$ ways that treatment labels could be assigned to the numbers in the top row. For each of these top row permutations, there are another 792 ways that treatment labels could be reassigned in the bottom row. Thus, there are 627,264 total permutations to consider. Of these, 6259 involve a treatment effect greater than or equal to the observed value, yielding a one-sided *p*-value of about 0.01. The exact same approach could be used with the Mann–Whitney test, in which case 11,577 permutations are greater than or equal to the observed test statistic, for a *p*-value of about 0.018.

How does this approach compare to conducting two separate tests, one for each gender group? Conducting separate Pitman tests for each gender group yields one-sided *p*-values of 0.12 for the male-only group, and 0.028 for the female-only group. This example illustrates the previously discussed problems with the multiple-comparison approach. Can the experimenter conclude, in this multiple-comparison exercise, that the null hypothesis of no treatment effect is rejected? Moreover, if the experimenter really was seeking to test every combination of treatment effect and gender grouping simultaneously, then the testing procedure should be constructed in a way that controls the family-wise error rate of these tests (the probability of falsely rejecting at least one null hypothesis among the two tests). While a detailed treatment of multiple-comparison adjustments is beyond the scope of this discussion, it is helpful to note that Bonferroni-adjusted *p*-values are 0.24 for the male-only group and 0.056 for the female-only group, substantially greater than the 0.01 value of the stratified test.^20^

Stratified permutation testing provides expositional simplicity and statistical power when nuisance variables are discrete. It does so by tailoring the permutation strategy to the underlying randomization of the experiment’s design. It bears emphasis that the null hypothesis for the stratified permutation test—that observations are drawn from the same distribution *within strata*—does not restrict observations to share a common distribution *across strata*. Distributions should be the same within strata, though, apart from potential differences in location. Treatment effects should also be the same across strata; otherwise, separate tests would be appropriate.^21^

Before moving on, note that this stratification process generalizes easily to situations with multiple nuisance variables. A simple illustration is provided by Comeig et al. (2022), who report an experiment designed to study risk appetite as a function of framing (“downside risk” vs “upside risk”), payoff scale, and subject gender. The experiment involved 256 subjects, half male and half female, each tasked with making a choice between a risky lottery and a safe lottery.^22^ Half of the subjects were exposed to treatments in which the risky option was presented as downside risk (a small probability of a low payoff); the other half were presented the risky option as upside risk (a small probability of a large payoff). If small probabilities are “over-weighted,” as prospect theory predicts, then subjects would tend to shy away from the downside risk of a low payoff and be attracted to the upside risk of a low probability of a high payoff. In every pairwise choice, both experimental lotteries were constructed so that the expected payoff from the risky lottery exceeded the payoff from the safe lottery by the same fixed amount. The standard deviations of the two lotteries were also the same. Finally, these treatments were blocked on payoff scale, with half of subjects presented payoffs five times larger than the other half. Of the 32 male and 32 female subjects exposed to each combination of risk profile and payoff scale, the number of subjects choosing the risky option is presented in Table 9, below.

**Table 9:** Risky option choice proportions by treatment

Gender	Payoff scale	Downside risk (%)	Upside risk (%)
Male	1×	25/32 = 78	30/32 = 94
Male	5×	17/32 = 53	28/32 = 88
Female	1×	19/32 = 59	29/32 = 91
Female	5×	4/32 = 13	27/32 = 84

Comeig et al. (2022): Table 2

While these data may be used to explore various hypotheses, perhaps the most interesting prediction is that subjects should be more willing to take upside risks than downside risks, even when the expected payoff and standard deviation of the safe and risky options are the same. In testing this hypothesis, both gender and payoff scale are nuisance variables.^23^

A stratified permutation test of the effect of risk type on lottery choice would involve permuting risk-type labels across each of the 256 lottery choices, subject to the constraint that labels are not reassigned across any of the strata in the different rows of the table. With two crossed nuisance variables, this equates to preserving four separate strata during the permutation process: male/1×, male/5×, female/1×, and female/5×. Otherwise, the procedure is the same as with one nuisance parameter.

Given the large number of possible permutations for a sample of this size, an exact permutation test would be computationally costly. An approximate permutation test can be conducted by randomly sampling many possible permutations, subject to stratification constraints, and comparing the test statistic values of these random draws against the observed value. Here, pooling across gender and payoff scale, 49 more subjects selected the risky lottery in the case of upside risk than in the case of downside risk. Over 999,999 random permutations of risk-type labels within the respective strata, none yielded a pooled difference between the upside and downside risk treatments as extreme as the observed difference, implying a *p*-value of less than 0.001. Similar tests, omitted here, could be used to evaluate the effects of payoff scale or subject gender.

One of the most attractive properties of this stratified permutation approach is the intuitive nature of randomizing across only the dimension of the data at focus. Indeed, the stratification tests we consider in this section are really just generalizations of the matched-pairs permutation strategy. In the matched-pairs context, each pair of observations is treated as its own stratum. In the more general stratified permutation testing context, multiple observations may fall within each stratum, and different strata may have different numbers of observations. The intuition behind stratified permutation also generalizes to other influences that might be accounted for in the permutation process (Ehlert et al., 2020; Heß, 2017). As a general strategy for conducting statistical inference in the presence of nuisance variables, stratified permutation testing strikes an attractive balance of analytical flexibility and ease of presentation.

### Stratified permutation tests for *k* > 2 treatments with nuisance variables

The idea of stratified permutation testing generalizes in an intuitive way to higher order comparisons with $k > 2$ treatments. As a concrete example, return to the Smith (1964) experiment, selected data from which is reproduced in Table 7. When using the Jonckheere–Terpstra test to look for an ordered treatment effect in Sect. 5.1, we relied on economic theory to justify pooling results across sessions with different group sizes: 20 traders in Group A sessions versus 24 traders in Group B. Instead of combining the data for the two rows of each treatment column as was done in the prior section, an alternative would be to treat the presence of extra-marginal units as a nuisance variable.

In other words, the rows of Table 7 can be seen as strata with a one average price observation for each of three treatments. In general, a stratified Jonckheere–Terpstra test would involve permuting observations across treatments but not across strata. Here, since there are no ties or reversals for treatment comparisons within a row of the table, the *p*-value can be computed analytically. There are $3!$ ways to permute the observations in each row, so there are $\left( {3!} \right)^{2} = 36$ possible permutations of the sample data that do not move observations across the separate strata. Since the most extreme outcome in the conjectured direction is what is observed, the *p*-value of a stratified Jonckheere–Terpstra test is $1/36 \approx 0.028$, only a little larger than the 0.022 *p-*value obtained when ignoring the nuisance variable. In contrast, running separate tests for each row would limit the researcher to minimum *p*-values of $1/3 \approx 0.333$ for each test.

The similarity of the previous example to the analysis of matched-pairs data is no coincidence. The matched-pair design (in which each subject is exposed to two treatment conditions) is a special case of a more general design in which a subject, or group of subjects, is exposed to $k > 2$ treatment conditions. In the language of classical statistics, the resulting data invite two-way analysis of variance: the experimenter may be primarily interested in the *k* treatment effects, but analysis also needs to account for dependence relationships among the multiple measurements taken from each subject or group.

A clean example of this *k*-treatment data structure is provided by Ma et al. (2022), who report a laboratory experiment designed to measure valuation of paintings with 6 different color configurations. In a laboratory setting, the authors showed 465 unique subjects sets of 6 constructed Rothko-like paintings, each with a different primary color (blue, red, green, yellow, etc.). Subjects were given an opportunity to purchase each painting under a bidding process that incentivized private-value revelation.^24^ Results indicated substantial differences in average valuation by color, with bids for red and blue paintings exceeding the average bid by about 17–19 percent.

With each subject viewing and bidding on multiple paintings, subject heterogeneity could be pronounced in this design. A subject who particularly liked art, or who happened to need a painting for decorative purposes, might bid higher on all 6 colors than another subject. If this experiment had involved only a few subjects, then a stratified permutation test for differences in valuation by color could be based upon the two-way ANOVA *F* statistic. For the null hypothesis of no treatment effect between any of the 6 colors, the observed *F* statistic could be compared to a null distribution generated by permuting color labels within each subject’s bids (controlling heterogeneity by constraining permutations never to cross subject-strata in the sample data). With a sample consisting of the price bids of 465 subjects, however, standard parametric tests are a better option.

Interestingly, a rank-based analogue of the permutation ANOVA test just described was long ago proposed by economist Milton Friedman (1937, 1939, 1940), who later won a Nobel Prize in Economics for other works. Friedman argued that normality is “likely to be the exception rather than the rule” when studying socio-economic data and devised a rank-based test because computations using ranks were “less arduous… requiring but a fraction of the time” when working with “large scale collections of social and economic data” in the years leading up to World War II. (Friedman, 1937: p. 675). Times have changed and, like other rank-based tests, the advantages of the Friedman test are now limited. Importantly, the difficulty of computing exact *p*-values under the Friedman test is the same as the difficulty of computing exact *p*-values for analogous ANOVA models based on measured observations.

## Permutation tests for linear relationships

A final class of statistical tests arises in situations where the experimenter has reason to expect a linear or linearizable relationship between the treatments and outcomes of interest. Sometimes this involves statistical inference around a measure of correlation. Sometimes it involves the parameters of multiple regression models. In both cases, permutation methods can provide a basis for small-sample statistical inference.

### Tests of correlation coefficients

Correlation studies arise with some frequency in experimental settings. The data in a correlation study consist of $n$ pairs of observations $\left\{ {\left( {x_{1} ,y_{1} } \right), \ldots ,\left( {x_{n} ,y_{n} } \right)} \right\}$. As explained in every basic course on applied statistics, the Pearson correlation coefficient, $r$, is a function of the products of deviations of the sample observations from their respective sample means:

11 $$r = \frac{{\mathop \sum \nolimits_{i = 1}^{n} \left( {x_{i} - \overline{x}} \right)\left( {y_{i} - \overline{y}} \right)}}{{\sqrt {\mathop \sum \nolimits_{i = 1}^{n} \left( {x_{i} - \overline{x}} \right)^{2} } \sqrt {\mathop \sum \nolimits_{i = 1}^{n} \left( {y_{i} - \overline{y}} \right)^{2} } }}$$

The null hypothesis of no correlation corresponds to a situation in which there is no monotonic relationship between the $x$ and $y$ values.

In conventional hypothesis testing, the observed value of the correlation coefficient, $r_{obs}$, would be compared to the null distribution of this statistic—something that could be inferred from assumptions about the distributions of the $x$ and $y$ values under the null hypothesis, or approximated via reference to a limit theorem for large enough sample sizes. As in the previously discussed locational tests, however, these approaches are difficult to defend for the small samples typical of many experiments.

Fortunately, permutation tests can be constructed around correlation coefficients. If there were no association between the variables, as the null hypothesis insists, then observed covariation would reflect exchangeable error in the experimental design. Because each observed value of the measured outcome would be equally likely to have been observed under every value of the treatment variable, the null distribution of the correlation test-statistic can be constructed by computing the value of $r$ under each of the $n!$ ways that one of the two variables could be reordered, holding the order of the other variable constant. For a two-sided test, the permutation *p*-value would be computed as follows:

12 $${\text{permutation}}\;{\text{test}}\;{\text{of}}\;{\text{correlation}},\;{\text{two-sided}}\;p{\text{-value}} = \frac{{\mathop \sum \nolimits_{i = 1}^{n!} 1\left( {\left| {r_{i} } \right| \ge \left| {r_{obs} } \right|} \right)}}{n!}$$

To illustrate, consider the traveler’s dilemma experiment reported by Capra et al. (1999). Subjects were randomly paired and tasked with making simultaneous “claims,” subject to the following rules. Each subject’s claim had to fall within the range from 80 to 200. After both claims were made, each subject would earn the smaller of the two claims, minus a penalty of *R* if the subject’s claim was the larger claim and plus a reward of *R* if the subject’s claim was the smaller claim. No penalty or reward was applied if the two claims were equal. Since each subject has a unilateral incentive to undercut anything greater than the minimum in this game, the unique Nash equilibrium is for both players to make the minimum claim regardless of the size of the incentive parameter. This prediction is counterintuitive. Common sense suggests that claims should fall with increases in the size of the penalty (for being high) and reward (for being low).

The experiment was conducted with session groups of 9–12 subjects, randomly matched into pairs to play 10 rounds of the game with one incentive parameter in Part A, followed by additional rounds with a different incentive parameter in Part B. There was one session for each pair of A and B treatments, as shown in Table 10. Here, we focus on the Part A data; we will turn to Part B in Sect. 7.2.

**Table 10:** Session-average claims for a traveler’s dilemma game

Treatments and results	S1	S2	S3	S4	S5	S6
*Part A*						
Incentive term (*R*)	80	10	50	20	25	5
Average claim	82	186	92	116	146	196
*Part B*						
Incentive term (*R*)	10	80	20	50	5	25
Average claim	163	99	86	82	171	170

Capra et al. (1999): average claims over final 5 rounds of treatment

The most salient feature of the Part A data in the top part of the table is the apparent sensitivity of claims to the size of the incentive parameter. Indeed, the Part A data appear more consistent with the intuitive prediction that average claims would vary inversely with the incentive parameter than with the Nash prediction of no effect. In fact, the observed value of the correlation between penalties and average claims in Part A is $r_{obs} = - 0.873$. To test whether that observation is statistically significant, the null distribution of the correlation coefficient can be constructed by computing the correlation coefficient under every possible permutation of the observed claims data. Of the $6! = 720$ possible permutations to be considered in this way, only 3 result in a correlation coefficient equal to or smaller than the observed value. Thus, a one-sided *p*-value for a permutation test of negative correlation against the null is $3/720 \approx 0.004,$ a significant rejection of the Nash hypothesis, despite the limited sample size.^25^

Before moving on, note that nothing about the permutation strategy just described depends on use of the Pearson correlation coefficient as the test statistic. The Pearson statistic made sense for the theoretically continuous data under investigation, but other experimental contexts could motivate the use of other statistics. In an “bomb” risk aversion experiment (Crosetto & Filippin, 2013), for example, a subject is shown 12 boxes and allowed to check as many boxes as desired, understanding that each box checked earns the subject $1 unless and until a randomly hidden bomb is encountered—in which case nothing is earned. This setup can be used to elicit risk aversion, but the mapping from number-of-boxes-checked to some measure of risk aversion depends on the measure of risk aversion used (e.g., constant relative risk aversion) and is nonlinear in any event. In this application, it might make sense to rank subjects by the number of boxes they choose to check and by some other proxy for risk aversion, such as amount saved for retirement. In this ordinal context, a rank-based measure of association—such as Kendall’s $$\tau$$ or Spearman’s $$\rho$$ statistic—would be a better correlation concept. The same approach described above could be used to arrive at a permutation *p*-value in this setting. The only difference would be the measure of association used as the test statistic.

### Tests of linear regression models

Moving beyond correlation analysis of a bivariate pattern, permutation methods can also be used to conduct statistical inference for linear models with more independent variables. There are, however, significant limitations to permutation inference in this setting. Obvious permutation strategies are forthcoming only for a few special regression models. In most cases, the experimenter will face a choice of different permutation strategies, and effort may be required to identify the appropriate strategy for the application. Standard parametric tests do not exhibit these difficulties and, for moderate sample sizes, may be robust enough to deviations from parametric assumptions to support credible inference. For small sample sizes, however, permutation tests will still constitute a more reliable basis for inference.

Starting with one of the lucky special cases for permutation testing, consider the following bivariate data generating process:

13 $${\varvec{y}} = \alpha + \delta {\varvec{z}} + \varvec{\epsilon}$$

For $\varvec{\epsilon}$ a mean-zero error term unrelated to the value of the regressor ${\varvec{z}}$. Suppose interest is in testing the null hypothesis $\delta = 0.$ Under the null hypothesis, all variation in the elements of ${\varvec{y}}$ is attributable to random error, ${\varvec{y}}_{0} = \alpha + \varvec{\epsilon}$, so every element of the ${\varvec{y}}$ vector is equally likely to have been paired with every element of the ${\varvec{z}}$ vector. This observation suggests a simple permutation strategy: compare the observed *t* statistic for the least squares estimate of $\delta$ against the set of *t* statistics calculated under all possible permutations of the order of elements in the ${\varvec{y}}$ vector while holding fixed the order of elements in the ${\varvec{z}}$ vector.^26^ This should look familiar. It is the permutation strategy for correlation coefficients described in Sect. 7.1.

As a concrete illustration, return to the Capra et al. (1999) traveler’s dilemma experiment, with data reproduced in Table 10. First consider a simple linear regression of average claim (${\varvec{y}}$) on penalty term (${\varvec{z}}$) using data from Part A of the experiment. Fitting the linear model ${\varvec{y}} = \alpha + \delta {\varvec{z}} + \varvec{\epsilon}$ via OLS yields the following parameter estimates and standard errors:

$$\begin{array}{*{20}r} \hfill {\hat{\alpha } = 182.917} & \hfill {\hat{\delta } = - 1.4711} \\ \hfill {\left( {16.814} \right)} & \hfill {\left( {0.411} \right)} \\ \end{array}$$

The *t* statistic for this estimate of $\delta$ is $t_{obs} = - 1.471/0.411 = - 3.581$. Under the null hypothesis that $\delta = 0$ (the size of the incentive parameter has no effect on average claims), all variation in the ${\varvec{y}}$ vector is attributable to exchangeable error. This means that the null distribution of the *t* statistic can be computed by permuting the order of the ${\varvec{y}}$ vector and recalculating the value of the *t* statistic at each permutation. With 6 observations, there are $6! = 720$ ways to permutate the order of elements in the ${\varvec{y}}$ vector under the null hypothesis. Of these 720 permutations, 12 yield *t* statistic values at least as extreme as $t_{obs}$, yielding a two-sided *p*-value of $12/720 \approx 0.017$ for the effect of the incentive parameter on average claims in Part A.

Next consider the same model applied to Part B. Fitting the same model via OLS when using the data from Part B of the experiment yields the following parameter estimates and standard errors:

$$\begin{array}{*{20}r} \hfill {\hat{\alpha } = 159.434} & \hfill {\hat{\delta } = - 0.977} \\ \hfill {\left( {24.345} \right)} & \hfill {\left( {0.595} \right)} \\ \end{array}$$

The *t* statistic for this estimate of $\delta$ is $t_{obs} = - 1.642$ and—by the same permutation process as above—a two-sided *p*-value for the effect of the incentive parameter on average claims in Part B is found to be $150/720 \approx 0.208$.

Visual examination of the Part A and Part B outcomes in each treatment column of Table 10 suggests the presence of order effects—that is, experiences in Part A seem like they could be introducing confounding variation in the Part B data. To explore this possibility, consider the more complicated linear model that allows for average claims in Part B of the experiment (${\varvec{y}}$) to depend on both the Part B incentive parameter (${\varvec{z}}$) and the nuisance influence captured by the average claim in Part A (${\varvec{x}}$). Fitting the multiple regression model ${\varvec{y}} = \alpha + \beta {\varvec{x}} + \delta {\varvec{z}} + \varvec{\epsilon}$ via OLS is trivial. Conducting permutation inference on this model is not.

To see why multiple regression with nuisance variables presents a harder problem, consider the general case of a data generating process with the following form:

14 $${\varvec{y}} = \varvec{X\beta } + \varvec{Z\delta } + \varvec{\epsilon}$$

For $\varvec{\epsilon}$ an error term as before, ${\varvec{X}}$ a matrix of nuisance variables which may include a constant term, and ${\varvec{Z}}$ a matrix of regressors of interest. Under the null hypothesis that ${\varvec{\delta}} = 0$, each element of the response vector is now more than random error: ${\varvec{y}}_{0} = \varvec{X\beta } + \varvec{\epsilon}$. This connection makes the simple permutation strategy of reordering ${\varvec{y}}$ generally indefensible. Variation in the ${\varvec{y}}$ vector is partly attributable to the influence of the nuisance variables in ***X***. One might initially think that this could be solved by either subtracting $\varvec{X\beta }$ from both sides of the equation or by permuting the rows of the ${\varvec{X}}$ matrix in lockstep with the rows of the ${\varvec{y}}$ vector but neither of these strategies is attractive. The first requires knowledge of ${\varvec{\beta}}$, which is unavailable in most interesting cases; the second fails to preserve collinearity between ${\varvec{X}}$ and ${\varvec{Z}}$.

In fact, while many permutation strategies have been suggested for the multiple regression context, there remains no generally accepted permutation approach for this problem. A full survey of this literature is beyond the scope of discussion, but helpful commentaries are provided by Kennedy (1995), Kennedy and Cade (1996), Manly (2007: ch. 8), Anderson and Robinson (2001), and Winkler et al. (2014). To illustrate one intuitive option, consider the following permutation strategy due to Freedman and Lane (1983):

1. Fit the full model by OLS, ${\varvec{y}} = \varvec{X\beta } + \varvec{Z\delta } + \varvec{\epsilon}$, and compute the observed *F* statistic for testing the null hypothesis that ${\varvec{\delta}} = 0$.
2. Fit the reduced model by OLS, ${\varvec{y}} = \varvec{X\beta } + \varvec{\epsilon}$, and use this model to compute a reduced-model prediction vector $\hat{\varvec{y}} = \varvec{X\hat{\beta }}$ and a reduced-model residual vector ${\varvec{r}} = {\varvec{y}} - \hat{\varvec{y}}$.^27^
3. Permute the order of the reduced-model residual vector $\varvec{r }$ and add each permutation of the residual vector to the reduced-model prediction vector $\hat{\varvec{y}}$ to generate a new permutation of the ${\varvec{y}}$ vector. For the observed data, this reconstructs the observed ${\varvec{y}}$ vector. For all other permutations, it constructs a new ${\varvec{y}}_{{\varvec{p}}}$ vector in which only the variation not explained by the nuisance variables is being permuted.
4. For each such permutation, fit the full model ${\varvec{y}}_{p} = \varvec{X\beta } + \varvec{Z\delta } + \varvec{\epsilon}$ and compute the *F* statistic for testing the null hypothesis that ${\varvec{\delta}} = 0$. For testing individual parameters, *t* statistics could be used instead.
5. Compare the observed value of the *F* statistic (or *t* statistic) from step 1 to the permutation distribution from step 4 to compute a *p*-value for this test.

This procedure can be made concrete by applying it to the multiple regression model described above for Part B of the Capra et al. (1999) data. Specifically, consider regressing average claim in Part B (${\varvec{y}}$) on both average claim in Part A (${\varvec{x}}$) and incentive term in Part B (${\varvec{z}}$). Fitting the model ${\varvec{y}} = \alpha + \beta {\varvec{x}} + \delta {\varvec{z}} + \varvec{\epsilon}$ via OLS yields the following parameter estimates and standard errors:

$$\begin{array}{*{20}r} \hfill {\hat{\alpha } = 93.300} & \hfill {\hat{\beta } = 0.589} & \hfill {\hat{\delta } = - 1.425} \\ \hfill {\left( {39.033} \right)} & \hfill {\left( {0.305} \right)} & \hfill {\left( {0.514} \right)} \\ \end{array}$$

For purposes of testing the joint null hypothesis that $\beta = \delta = 0$, the simple approach used in univariate regression could be repeated using the *F* statistic as a test statistic. For testing the less restrictive null that $\delta = 0,$ a more involved permutation scheme is needed.

Following the Freedman and Lane (1983) procedure outlined above, we can start by noting the observed *t* statistic value of $t_{obs} = - 1.425/0.514 = - 2.773$ for the estimate of $\delta$. Fitting the reduced model ${\varvec{y}} = \alpha + \beta {\varvec{x}} + \varvec{\epsilon}$ via OLS then yields the two components needed to construct approximate permutations of the ${\varvec{y}}$ vector: (1) a vector of predicted values from this reduced regression and (2) a vector of residuals from the reduced regression. To construct the null distribution, we permute the order of elements in the residual vector, each time adding it to the predicted value vector to form a new permutation of the ${\varvec{y}}$ vector, and then fit that new ${\varvec{y}}$ vector to the full model, recording the *t* statistic associated with the estimated value of $\delta$. Of the 720 permutations of the *t* statistic computed in this manner, 35 yield *t* statistics with equal or greater absolute value than $t_{obs}$, resulting in a two-sided test *p*-value of about $0.049$. This result is consistent with the guess that order effects could be muddying relationships in the Part B data.

## Conclusion

Two themes have emerged throughout this survey of permutation methods. The first is that individual permutation tests are best understood not as idiosyncratic elements of a loosely related set of tests but as special cases of a general approach to conducting statistical inference when working with experimental data. The second theme is that permutation tests that operate on measured data will generally provide a more intuitive and defensible basis for inference than those based on rank-transformed data, at least where observations are measured as interval data. Both themes are reflected in the structure of Table 1.

The rows of Table 1 illustrate the common approach that underlies all permutation tests. Starting from the experimental design and the null hypothesis, the researcher first locates the appropriate permutation strategy, then selects a test statistic with power to detect the alternative hypothesis of interest. Statistical inference is conducted by comparing the observed value of the test statistic against the empirical null distribution of that test statistic under the permutation strategy. Every permutation test is an application of this same process.

The columns of Table 1 illustrate the opportunity cost of what we consider overreliance on familiar rank-based tests. Every popular rank-based test is simply the application of a more general test to rank-transformed values of the measured data. When applied to interval data, the rank transformation discards potentially important sample information. While this may have consequences for statistical power in some applications, we rest our critique on the more basic point that the rank transformation is unintuitive and unnecessary in most cases. Rank-based permutation tests may be justified by properties of the experimental design or the data that has been collected but should not be the unquestioned default choice that they are today.

Table 1 also illustrates how the appropriate choice of permutation approach depends on the treatment structure of the experiment. The flip side of this is the importance of designing an experiment to shine a bright light on the research questions of interest. Experimentalists should consider going beyond the standard treatment-and-control framework. An attractive alternative is the use of intensity-based treatments to generate *k* ordered samples, which can be evaluated with the Directional Difference Test listed in the fourth row of Table 1. This approach can yield large gains in sensitivity to treatment effects even with small sample sizes.

In addition, the stratified permutation tests shown in the fifth row of Table 1 offer two important advantages. The first is an opportunity to eschew the complexity and power-loss of multiple-comparison corrections in some settings. The second is the ability to conduct coherent analysis of rich experiment designs by focusing on treatment variations, one at a time, without losing track of nuisance variable differences between strata. Since the presence of nuisance variables (from procedural variations or secondary treatments) is more the rule than the exception for experiments in the social sciences, the ability of permutation tests to accommodate stratification is likely to be one of the most useful features of this methodology.

As our discussion illustrates, permutation testing moves fluidly between experimental design and data analysis. Experimenters can select and customize permutation tests to fit specific design choices and research goals, just as they can design experiments with expected permutation tests in mind. This type of bespoke hypothesis testing requires more thought—and often more application-by-application scripting—than conventional testing based on familiar, prepackaged routines. We think the game is worth the candle. Our hope is that this discussion inspires a richer use of the range of permutation tests available to experimental researchers, and a stronger and more efficient use of the data we collect.

## Supplementary Information

Below is the link to the electronic supplementary material.

Supplementary file1 (PDF 92 kb)

Supplementary file2 (PDF 122 kb)

## Footnotes

1 For example, Kagel and Roth (2000) summarize a comparison of different “clearinghouse” methods of matching medical residents with hospitals in the United Kingdom. Two locations used a “priority product” method that tended to fail; two locations used a “deferred acceptance” method that, in theory, was “stable” with respect to bilateral deviations from assigned matches; and one location switched from one method to the other. Minor procedural and geographic details differentiated the locations and the match values and costs of making early matches were unobserved, so the authors conducted a laboratory experiment in which each of the alternative matching methods was used in three laboratory sessions with carefully crafted parallel conditions.

2 Siegel’s impact on experimental methodology cannot be overstated. Indeed, the annual Economic Science Association prize for the best experimental economics dissertation is still called the *Sidney Siegel Award*.

3 The published paper analyzed the difference in price averages with a *t* test but also reported a Mann–Whitney test for the difference in price medians (Caginalp et al., 1998).

4 As for most topics in statistics, R. A. Fisher also has strong claim to name recognition for this approach (e.g., Fisher 1936). Manly (2007: p. 113) provides an interesting discussion of philosophical contrasts between Pitman’s and Fisher’s permutation arguments. Somewhat a reflection of the weight of each scholar’s work, but mainly for expositional clarity, we refer to permutation testing in the two-independent-sample context as a Pitman permutation test, and to permutation testing in the matched-sample context as a Fisher permutation test. Miller (1997: pp. 27, 53) adopts this same convention.

5 If simulations are used, it is advisable to run several large simulations to be sure that the resulting *p* value proportions are not affected in terms of the number of trailing digits being reported.

6 The authors used a Pitman permutation test with the peak price data shown in Table 4 above (Bohr, Holt, & Schubert, 2019).

7 The sharper result obtained with the Pitman test owes to the fact that the “reversals” from the general trend (peaks of 43, 42.5 and 38 under the government-savings treatment) are only slightly larger than some of the more modest peaks under the private-savings treatment (42, 38.5 and 36), whereas the largest peak prices under the private-savings treatment (70, 61.5, and 53) greatly exceed most of the government-savings observations.

8 Holt and Smith (2022) employ stratified permutation testing to evaluate these data (discussed in Part 6), with a secondary strata for group size.

9 For example, measures of individual characteristics, e.g. risk aversion or type-A personality, are typically considered to be ordinal in the absence of precise preference models that may observed responses to questions.

10 Specifically, Moir (1998) finds the Pitman test (in that paper referred to as the “ER means test”) to perform about as well as the t-test when the underlying distribution is close to normal and to outperform the *t*-test in some non-normal settings. The Mann–Whitney test underperformed both the Pitman test and *t*-test in most settings considered.

11 Specifically, in the Power design, the mixed strategy Nash equilibrium price distributions are above the competitive price, and in the No-Power design the Nash equilibrium price equals the competitive price.

12 Constant market power is achieved by structuring demand and cost conditions such that the symmetric mixed strategies of sellers in setting prices yields the same predicted price distributions for each treatment.

13 Davis and Holt (1994) based their analysis on standard Wilcoxon signed-rank tests.

14 Of course, $\overline{d} = \overline{x} - \overline{y}$, so the test statistic could equivalently be expressed as in Eq. (1). We adopt this form of the test statistic because it better matches the intuition of the matched-pairs permutation strategy.

15 This description omits an important feature of the UK auction that permits lease winners to bid in a second-phase reverse auction (low bids win) to sell electricity back to a government agency at a guaranteed price corresponding to their successful reverse auction bid.

16 The “extra” traders in the 24-person sessions had extra-marginal units to the right of the supply–demand intersection. Equilibrium price and quantity predictions were thus unaffected by the presence of these traders.

17 Some of the reported tests were based in multiple price observations within a session. Here we stick to the more conservative practice of using session-level measurements to ensure that observations are independent.

18 An alternative is to construct a permutation test based on the ranks of binary differences (Shan, Young, & Kang, 2014).

19 Holt, Porzio, and Song (2017) employed a Pitman test, stratified by gender, to assess this treatment effect.

20 Bonferroni corrections are one of several possible strategies for addressing what is often called the “multiple comparisons problem.” Miller (1997: p. 75) provides a gentle introduction to the subject. List, Shaikh, and Xu (2019) provide a more detailed treatment in the experimental context. Example applications in experimental work include Holt et al. (2012) and Smerdon, Offerman, and Gneezy (2020).

21 Put another way, the maintained assumption is that the shift model applies within each strata and that all shifts are of the same magnitude across strata.

22 The paper also contains other data for 10 choice pairs, with one selected at random ex post for payment. Here, we restrict attention to the single-choice data, i.e. a single pair of upside or downside risk choices that was used for the treatments in which each subject only made a single decision.

23 Comeig et al. (2022) employed a Pitman permutation test, stratified by gender and payoff scale to assess this treatment effect.

24 Bids were solicited using the Becker, DeGroot, Marschak (1964) method.

25 Capra et al. (1999) report the *p*-value of an improvised permutation test based on counts of possible reversals between treatments with adjacent incentive parameters.

26 In the one-regressor context, the value of the parameter estimate is also a suitable test statistic (instead of the *t* statistic). This does not hold true for more complicated models.

27 We abuse notation for simplicity in this section. The unobserved error vector $({\varvec{\epsilon}}$) and parameter values will differ between one specification of the model and the next.
