# Supplementary material for: Permutation tests for experimental data
Source: Exp Econ. 2023 Apr 1:1–38. Online ahead of print. doi: 10.1007/s10683-023-09799-6 (PMC10066020; doi:10.1007/s10683-023-09799-6)
Supplement: Supplementary file 1 — Supplementary file1 (PDF 92 kb) [file 10683_2023_9799_MOESM1_ESM.pdf]

Appendix A

Tools for Permutation Testing

This appendix collects resources for experimenters interested in running permutation tests but uncertain what tools best suit their workflow. All tests described in Holt and Sullivan (2023) were performed using R, with code provided in Appendix B. Several tests are made available for public use through a web portal at http://veconlab.econ.virginia.edu/rand/rand.php.

Other sources of instruction on permutation testing and coding include Basso, Pesarin, Salmaso, and Solari (2009) (example R code), Good (2005) (brief illustrations from “Resampling Stats,” R, C++, StatXact), Heß (2017) (“ritest” package for Stata), and Kaiser (2007) (“permtest1” and “permtest1” commands written for Stata). Example libraries and commands for writing and running permutation tests in different software packages are collected below.

Tools for permutation testing in R

Libraries with prepackaged permutation tests:

- `perm` - https://cran.r-project.org/web/packages/perm/index.html
- `lmPerm` - https://cran.r-project.org/web/packages/lmPerm/index.html

Libraries with routines for permuting data:

- `gtools` - https://cran.r-project.org/web/packages/gtools/index.html

Tools for permutation testing in Stata

Packages with prepackaged permutation tests:

- `ritest` - https://github.com/simonheb/ritest
- `permtest1` and `permtest2` - user-written Stata add-ons

Routines for permuting data:

- `permute` with `enumerate` option to conduct exhaustive permutation and `strata(svar)` option to stratify permutations - https://www.stata.com/manuals/rpermute.pdf

Tools for permutation testing in Python

Libraries with prepackaged permutation tests:

- `PyPermut` - https://github.com/qbarthelemy/PyPermut

Libraries with routines for permuting data:

- `itertools` - https://docs.python.org/3/library/itertools.html

Tools for permutation testing in other programs

Excel

- `resampling stats` add-in (commercial product) available at https://resample.com

Matlab

- `perms` - https://www.mathworks.com/help/matlab/ref/perms.html
- `randperm` - https://www.mathworks.com/help/matlab/ref/randperm.html

SAS

- ` ranperm` function - https://blogs.sas.com/content/iml/2013/09/23/generate-permutations-in-sas.html

Online

- `Permutation test` VeconLab program - http://veconlab.econ.virginia.edu/rand/rand.php

References

Basso, D., Pesarin, F., Salmaso, L., & Solari, A. (2009). *Permutation Tests for Stochastic Ordering and ANOVA*. Springer.

Good, P. (2005). *Permutation, Parametric, and Bootstrap Tests of Hypotheses* (3rd ed.). Springer.

Heß, S. (2017). Randomization inference with Stata: A guide and software. *Stata Journal*, 17(3): 630–651.

Kaiser, J. (2007). An exact and a monte carlo proposal to the Fisher–Pitman permutation tests for paired replicates and for independent samples. *Stata Journal*, 7(3): 402–412.
