# Supplementary material for: Permutation tests for experimental data
Source: Exp Econ. 2023 Apr 1:1–38. Online ahead of print. doi: 10.1007/s10683-023-09799-6 (PMC10066020; doi:10.1007/s10683-023-09799-6)
Supplement: Supplementary file 2 — Supplementary file2 (PDF 122 kb) [file 10683_2023_9799_MOESM2_ESM.pdf]

Appendix B

R Code for All Tests Described in Holt & Sullivan (2023)

```
# -------------------------------------------------------------------------
# Appendix for Holt & Sullivan (2023)
#
# The following R code demonstrates construction and computation of tests
# described in Holt & Sullivan (2023). This treatment gives preference to
# legibility, intuition, and ease of manipulation--at the cost of some
# repeated code blocks and poorly-optimized computational efficiency.
#
# Scripted for R version 4.2.2
# -------------------------------------------------------------------------



# Preamble ----------------------------------------------------------------

# Load libraries
require(tidyverse)
require(gtools)
require(vecsets)

# Set float tolerance
float.tol <- 10^(-10)

# Recursive function for generating k>2 null sample index
make_null_index <- function(this_group, group_sizes, constructed=NULL) {

  n_groups = length(group_sizes)
  n_values = sum(group_sizes)
  index_values = 1:n_values

  if (this_group == n_groups) {

    # calculate lengths
    this_group_size = group_sizes[this_group]

    # create matrix of new combinations, equal to whatever is left
    #new_combinations <- apply(constructed, MARGIN=1, function(row) {
    #  return( setdiff(index_values,row) )
    #}) %>% t()

    # create matrix of new combinations, equal to whatever is left
    new_combinations <- apply(constructed, MARGIN=1, function(row) {
      return( setdiff(index_values,row) )
    }) %>% matrix(ncol=group_sizes[this_group],byrow = TRUE)

    # return final construction
    return (cbind(constructed, new_combinations))

  } else if (this_group > 1) {

    # calculate lengths
    this_group_size = group_sizes[this_group]
    this_space_size = n_values - dim(constructed)[2]
    constructed_wid = dim(constructed)[2]
    constructed_len = dim(constructed)[1]
    combination_len = choose(this_space_size, this_group_size)

    # create array of new combinations for each row of constructed sample so far
    new_combinations <- apply(constructed, MARGIN=1, function(row) {
```

```
      remaining_index_values <- setdiff(index_values,row)
      return(
        combinations(this_space_size, this_group_size, remaining_index_values) %>%
          list()
      )
    })

    # melt new array into the combinations matrix
    next_constructed <- matrix(
      nrow = ( constructed_len *  combination_len),
      ncol = ( constructed_wid + this_group_size)
    )
    for (i in 1:constructed_len) {
      next_constructed[ ((i-1)*combination_len+1):(i*combination_len) , ] <-
        cbind( constructed[i,] %>%
                 rep(combination_len) %>%
                 matrix(nrow=combination_len,byrow=TRUE),
               new_combinations[[i]][[1]] )
    }

    # recurse
    next_group <- this_group + 1
    make_null_index(next_group, group_sizes, next_constructed)

  } else { # this_group = 1

    # calculate lengths
    this_group_size = group_sizes[this_group]

    # create matrix of combinations for first group
    new_combinations <- combinations(n_values, this_group_size, index_values)
    next_constructed <- new_combinations

    # recurse
    next_group <- this_group + 1
    make_null_index(next_group, group_sizes, next_constructed)

  }

}



# Data --------------------------------------------------------------------

# Table 2 (Caginalp, Porter, and Smith (1998): selected data from Table 2.)
tbl_2 <- list()
tbl_2$cash_rich <- c(3.43, 3.73, 3.97)
tbl_2$asset_rich <- c(3.03, 3.32, 2.55, 3.06)

# Table 4 (Bohr, Holt, and Schubert (2019): selected data from Table 1.)
tbl_4 <- list()
tbl_4$private_savings <- c(42, 36, 53, 61.5, 38.5, 70)
tbl_4$government_savings <- c(42.5, 21.25, 30, 26, 43, 38)

# Holt and Smith (2022)
dta_hs <- list()
dta_hs$low <- c(77, 83, 130, 81)
dta_hs$high <- c(132, 111, 93, 87)

# Table 5 (Davis and Holt (1994): Table 1, page 479.)
tbl_5 <- list()
tbl_5$c1_five_nopower <- c(329, 308, 341, 410, 310, 397)
tbl_5$c1_five_power <- c(407, 468, 430, 455, 397, 441)
tbl_5$c2_five_power <- c(415, 471, 392, 401, 392, 512)
tbl_5$c2_three_power <- c(425, 470, 408, 436, 424, 517)

# Table 6 (Holt & Sprott (2022): data on file with authors.)
```

```
tbl_6 <- list()
tbl_6$treatment <- c(rep("UK",4), rep("US",4), rep("Single",4))
tbl_6$levels <- c(rep(1,4), rep(2,4), rep(3,4)) %>% factor()
tbl_6$earnings <- c( -0.61, -3.64, 0.82, 1.39, 3.84, 1.04, 3.17,
  3.97, 0.22, -0.24, 0.42, 1.13 )

# Table 7 (Smith (1964): selected data from Table III.)
tbl_7 <- list()
tbl_7$treatment <- c("Rs","Rs","Rsb","Rsb","Rb","Rb")
tbl_7$levels <- c(1, 1, 2, 2, 3, 3) %>% factor()
tbl_7$group_size <- c(1, 2, 1, 2, 1, 2) %>% factor()
tbl_7$price <- c(208, 195, 213, 209, 217, 213)

# Table 8 (Holt, Porzio, and Song (2017): selected data from Tables 1 and 5.)
tbl_8 <- list()
tbl_8$female_25 <- c(87, 95, 61, 177.5, 75.5, 37, 152)
tbl_8$female_15 <- c(66, 36, 58, 64, 42)
tbl_8$male_25 <- c(55, 48, 68,  85,  65,  56.5, 50)
tbl_8$male_15 <- c(50, 70, 45, 43, 53)

# Table 9 (Comeig et al. (2022): Table 2.)
tbl_9 <- list()
tbl_9$male_1x_downside <- 1*(1:32<=25)
tbl_9$male_5x_downside <- 1*(1:32<=17)
tbl_9$male_1x_upside <- 1*(1:32<=30)
tbl_9$male_5x_upside <- 1*(1:32<=28)
tbl_9$female_1x_downside <- 1*(1:32<=19)
tbl_9$female_5x_downside <- 1*(1:32<=4)
tbl_9$female_1x_upside <- 1*(1:32<=29)
tbl_9$female_5x_upside <- 1*(1:32<=27)

# Table 10 (Capra et al. (1999): average claims over final 5 rounds of treatment.)
tbl_10 <- list()
tbl_10$R_A <- c(80,10,50,20,25,05)
tbl_10$claim_A <- c(82,186,92,116,146,196)
tbl_10$R_B <- c(10,80,20,50,05,25)
tbl_10$claim_B <- c(163,99,86,82,171,170)




# Section 2.1 -------------------------------------------------------------

#
# Pitman permutation test, data from Table 2
#

# Assign generic variables
x <- tbl_2$cash_rich
y <- tbl_2$asset_rich
z <- c(x,y)

# Print and store observed test statistic
obs_t <- mean(x) - mean(y)
obs_t

# Compute length of all samples
n_x <- length(x)
n_y <- length(y)
n_z <- length(z)

# Generate permutation index
perm_index_x <- combinations(n_z, n_x, 1:n_z)
perm_index_y <- apply(perm_index_x, 1, vecsets::vsetdiff, x=1:n_z) %>% t()

# Generate null sample distributions
null_x <- apply(perm_index_x, 1, function(lamda) {z[lamda]}) %>% t()
null_y <- apply(perm_index_y, 1, function(lamda) {z[lamda]}) %>% t()
```

```
# Generate null test statistic distribution and compute length
null_t <- apply(null_x, 1, mean) - apply(null_y, 1, mean)
n_null <- length(null_t)

# Print components of two-sided test
n_null
sum( abs(null_t) - abs(obs_t) >= -float.tol)
( sum( abs(null_t) - abs(obs_t) >= -float.tol)/n_null ) %>% round(3)

# Print components of one-sided test
n_null
sum( null_t - obs_t >= -float.tol)
( sum( null_t - obs_t >= -float.tol)/n_null ) %>% round(3)

# Print conventional t test
t.test(x, y, alternative="two.sided")$p.value %>% round(3)

# Generate Table 2
cbind(null_x,null_y,round(null_t,2)) %>%
  as_tibble(.name_repair = "unique") %>%
  arrange(null_t) %>%
  print.data.frame()

# Function outputing two-sided p-value of Pitman Permutation Test
pppt <- function(arg_x,arg_y) {
  fun_x <- arg_x
  fun_y <- arg_y
  fun_z <- c(fun_x,fun_y)
  fun_n_x <- length(fun_x)
  fun_n_y <- length(fun_y)
  fun_n_z <- length(fun_z)
  fun_obs_t <- mean(fun_x) - mean(fun_y)
  fun_perm_index_x <- combinations(fun_n_z, fun_n_x, 1:fun_n_z)
  fun_perm_index_y <- apply(fun_perm_index_x, 1, vecsets::vsetdiff, x=1:fun_n_z) %>% t()
  fun_null_x <- apply(fun_perm_index_x, 1, function(lamda) {fun_z[lamda]}) %>% t()
  fun_null_y <- apply(fun_perm_index_y, 1, function(lamda) {fun_z[lamda]}) %>% t()
  fun_null_t <- apply(fun_null_x, 1, mean) - apply(fun_null_y, 1, mean)
  fun_n_null <- length(fun_null_t)
  result <- sum( abs(fun_null_t) - abs(fun_obs_t) >= -float.tol)/fun_n_null
  return(result)
}

# Search for confidence interval lower bound
search_bound <- mean(x) - mean(y)
search_step <- 0.0005
search_p <- 1
while(search_p >= 0.1) {
  search_bound <- round(search_bound - search_step, 4)
  search_p <- pppt(x,(y+search_bound))
}
ci_lower <- search_bound + search_step

# Search for confidence interval upper bound
search_bound <- mean(x) - mean(y)
search_step <- 0.0005
search_p <- 1
while(search_p >= 0.1) {
  search_bound <- round(search_bound + search_step, 4)
  search_p <- pppt(x,(y+search_bound))
}
ci_upper <- search_bound - search_step

# Print confidence interval
c(ci_lower,ci_upper) %>% round(3)

# Print conventional t test confidence interval
t.test(x, y, alternative="two.sided", conf.level = 0.9)$conf.int %>% round(3)

# Clear variables
```

```
rm(n_null,n_x,n_y,n_z,null_x,null_y,null_t,obs_t,x,y,z,perm_index_x,
   perm_index_y,pppt,search_bound,search_step,search_p,ci_lower,ci_upper)




# Section 2.2 ------------------------------------------------------------

#
# Mann-Whitney test, data from Table 2
#

# Assign generic variables
x <- tbl_2$cash_rich
y <- tbl_2$asset_rich
z <- c(x,y)

# Compute length of all samples
n_x <- length(x)
n_y <- length(y)
n_z <- length(z)

# Generate ranked variables
z_ranked <- rank(z)
x_ranked <- z_ranked[1:n_x]
y_ranked <- z_ranked[-(1:n_x)]

# Print and store observed test statistic
obs_t <- mean(x_ranked) - mean(y_ranked)
obs_t

# Generate null sample distributions
null_x <- combinations(n_z, n_x, z_ranked, set=FALSE)
null_y <- apply(null_x, 1, vecsets::vsetdiff, x=z_ranked) %>% t()

# Generate null test statistic distribution and compute length
null_t <- apply(null_x, 1, mean) - apply(null_y, 1, mean)
n_null <- length(null_t)

# Print components of two-sided test
n_null
sum( abs(null_t) - abs(obs_t) >= -float.tol)
( sum( abs(null_t) - abs(obs_t) >= -float.tol)/n_null ) %>% round(3)

# Print components of one-sided test
n_null
sum( null_t - obs_t >= -float.tol)
( sum( null_t - obs_t >= -float.tol)/n_null ) %>% round(3)

# Function outputing two-sided p-value of Mann-Whitney Test
pmwt <- function(arg_x,arg_y) {
  fun_x <- rank(c(arg_x,arg_y))[1:length(arg_x)]
  fun_y <- rank(c(arg_x,arg_y))[-(1:length(arg_x))]
  fun_z <- c(fun_x,fun_y)
  fun_n_x <- length(fun_x)
  fun_n_y <- length(fun_y)
  fun_n_z <- length(fun_z)
  fun_obs_t <- mean(fun_x) - mean(fun_y)
  fun_perm_index_x <- combinations(fun_n_z, fun_n_x, 1:fun_n_z)
  fun_perm_index_y <- apply(fun_perm_index_x, 1, vecsets::vsetdiff, x=1:fun_n_z) %>% t()
  fun_null_x <- apply(fun_perm_index_x, 1, function(lamda) {fun_z[lamda]}) %>% t()
  fun_null_y <- apply(fun_perm_index_y, 1, function(lamda) {fun_z[lamda]}) %>% t()
  fun_null_t <- apply(fun_null_x, 1, mean) - apply(fun_null_y, 1, mean)
  fun_n_null <- length(fun_null_t)
  result <- sum( abs(fun_null_t) - abs(fun_obs_t) >= -float.tol)/fun_n_null
  return(result)
}

# Search for confidence interval lower bound
```

```
search_bound <- mean(x) - mean(y)
search_step <- 0.0005
search_p <- 1
while(search_p >= 0.1) {
  search_bound <- round(search_bound - search_step, 4)
  search_p <- pmwt(x,(y+search_bound))
}
ci_lower <- search_bound + search_step

# Search for confidence interval upper bound
search_bound <- mean(x) - mean(y)
search_step <- 0.0005
search_p <- 1
while(search_p >= 0.1) {
  search_bound <- round(search_bound + search_step, 4)
  search_p <- pmwt(x,(y+search_bound))
}
ci_upper <- search_bound - search_step

# Print confidence interval
c(ci_lower,ci_upper) %>% round(4)

# Clear variables
rm(n_null,n_x,n_y,n_z,null_x,null_y,null_t,obs_t,x,y,z,x_ranked,y_ranked,
   z_ranked,pmwt,search_bound,search_step,search_p,
   ci_lower,ci_upper)


#
# Pitman permutation test, data from Table 4
#

# Assign generic variables
x <- tbl_4$private_savings
y <- tbl_4$government_savings
z <- c(x,y)

# Print and store observed test statistic
obs_t <- mean(x) - mean(y)
obs_t

# Compute length of all samples
n_x <- length(x)
n_y <- length(y)
n_z <- length(z)

# Generate permutation index
perm_index_x <- combinations(n_z, n_x, 1:n_z)
perm_index_y <- apply(perm_index_x, 1, vecsets::vsetdiff, x=1:n_z) %>% t()

# Generate null sample distributions
null_x <- apply(perm_index_x, 1, function(lamda) {z[lamda]}) %>% t()
null_y <- apply(perm_index_y, 1, function(lamda) {z[lamda]}) %>% t()

# Generate null test statistic distribution and compute length
null_t <- apply(null_x, 1, mean) - apply(null_y, 1, mean)
n_null <- length(null_t)

# Print components of two-sided test
n_null
sum( abs(null_t) - abs(obs_t) >= -float.tol)
( sum( abs(null_t) - abs(obs_t) >= -float.tol)/n_null ) %>% round(3)

# Clear variables
rm(n_null,n_x,n_y,n_z,null_x,null_y,null_t,obs_t,x,y,z,perm_index_x,
   perm_index_y)


#
```

```
# Mann-Whitney test, data from Table 4
#

# Assign generic variables
x <- tbl_4$private_savings
y <- tbl_4$government_savings
z <- c(x,y)

# Compute length of all samples
n_x <- length(x)
n_y <- length(y)
n_z <- length(z)

# Generate ranked variables
z_ranked <- rank(z)
x_ranked <- z_ranked[1:n_x]
y_ranked <- z_ranked[-(1:n_x)]

# Print and store observed test statistic
obs_t <- mean(x_ranked) - mean(y_ranked)
obs_t

# Generate null sample distributions
null_x <- combinations(n_z, n_x, z_ranked, set=FALSE)
null_y <- apply(null_x, 1, vecsets::vsetdiff, x=z_ranked) %>% t()

# Generate null test statistic distribution and compute length
null_t <- apply(null_x, 1, mean) - apply(null_y, 1, mean)
n_null <- length(null_t)

# Print components of two-sided test
n_null
sum( abs(null_t) - abs(obs_t) >= -float.tol)
( sum( abs(null_t) - abs(obs_t) >= -float.tol)/n_null ) %>% round(3)

# Clear variables
rm(n_null,n_x,n_y,n_z,null_x,null_y,null_t,obs_t,x,y,z,x_ranked,y_ranked,
   z_ranked)


#
# Pitman permutation test, data from Holt and Smith (2022)
#

# Assign generic variables
x <- dta_hs$low
y <- dta_hs$high
z <- c(x,y)

# Print and store observed test statistic
obs_t <- mean(x) - mean(y)
obs_t

# Compute length of all samples
n_x <- length(x)
n_y <- length(y)
n_z <- length(z)

# Generate permutation index
perm_index_x <- combinations(n_z, n_x, 1:n_z)
perm_index_y <- apply(perm_index_x, 1, vecsets::vsetdiff, x=1:n_z) %>% t()

# Generate null sample distributions
null_x <- apply(perm_index_x, 1, function(lamda) {z[lamda]}) %>% t()
null_y <- apply(perm_index_y, 1, function(lamda) {z[lamda]}) %>% t()

# Generate null test statistic distribution and compute length
null_t <- apply(null_x, 1, mean) - apply(null_y, 1, mean)
n_null <- length(null_t)
```

```
# Print components of two-sided test
n_null
sum( abs(null_t) - abs(obs_t) >= -float.tol)
( sum( abs(null_t) - abs(obs_t) >= -float.tol)/n_null ) %>% round(3)

# Clear variables
rm(n_null,n_x,n_y,n_z,null_x,null_y,null_t,obs_t,x,y,z,perm_index_x,
   perm_index_y)


#
# Mann-Whitney test, data from Table 4
#

# Assign generic variables
x <- dta_hs$low
y <- dta_hs$high
z <- c(x,y)

# Compute length of all samples
n_x <- length(x)
n_y <- length(y)
n_z <- length(z)

# Generate ranked variables
z_ranked <- rank(z)
x_ranked <- z_ranked[1:n_x]
y_ranked <- z_ranked[-(1:n_x)]

# Print and store observed test statistic
obs_t <- mean(x_ranked) - mean(y_ranked)
obs_t

# Generate null sample distributions
null_x <- combinations(n_z, n_x, z_ranked, set=FALSE)
null_y <- apply(null_x, 1, vecsets::vsetdiff, x=z_ranked) %>% t()

# Generate null test statistic distribution and compute length
null_t <- apply(null_x, 1, mean) - apply(null_y, 1, mean)
n_null <- length(null_t)

# Print components of two-sided test
n_null
sum( abs(null_t) - abs(obs_t) >= -float.tol)
( sum( abs(null_t) - abs(obs_t) >= -float.tol)/n_null ) %>% round(3)

# Clear variables
rm(n_null,n_x,n_y,n_z,null_x,null_y,null_t,obs_t,x,y,z,x_ranked,y_ranked,
   z_ranked)




# Section 3.1 -------------------------------------------------------------

#
# Fisher permutation test, data from Table 5, contrast 1
#

# Assign generic variables
d <- tbl_5$c1_five_power - tbl_5$c1_five_nopower

# Print and store observed test statistic
obs_t <- mean(d)
obs_t

# Compute length of all samples
n_d <- length(d)
```

```
# Generate null sample distributions
signs <- c(-1,1)
null_signs <- permutations(2, n_d, signs, set=FALSE, repeats.allowed=TRUE)
null_d <- sweep(null_signs,2,abs(d),`*`)

# Generate null test statistic distribution and compute length
null_t <- apply(null_d, 1, mean)
n_null <- length(null_t)

# Print components of two-sided test
n_null
sum( abs(null_t) - abs(obs_t) >= -float.tol)
( sum( abs(null_t) - abs(obs_t) >= -float.tol)/n_null ) %>% round(3)

# Print components of one-sided test
n_null
sum( null_t - obs_t >= -float.tol)
( sum( null_t - obs_t >= -float.tol)/n_null ) %>% round(3)

# Clear variables
rm(n_null,n_d,null_signs,null_d,null_t,obs_t,signs,d)


#
# Fisher permutation test, data from Table 4, contrast 2
#

# Assign generic variables
d <- tbl_5$c2_three_power - tbl_5$c2_five_power

# Print and store observed test statistic
obs_t <- mean(d)
obs_t

# Compute length of all samples
n_d <- length(d)

# Generate null sample distributions
signs <- c(-1,1)
null_signs <- permutations(2, n_d, signs, set=FALSE, repeats.allowed=TRUE)
null_d <- sweep(null_signs,2,abs(d),`*`)

# Generate null test statistic distribution and compute length
null_t <- apply(null_d, 1, mean)
n_null <- length(null_t)

# Print components of two-sided test
n_null
sum( abs(null_t) - abs(obs_t) >= -float.tol)
( sum( abs(null_t) - abs(obs_t) >= -float.tol)/n_null ) %>% round(3)

# Print components of one-sided test
n_null
sum( null_t - obs_t >= -float.tol)
( sum( null_t - obs_t >= -float.tol)/n_null ) %>% round(3)

# Clear variables
rm(n_null,n_d,null_signs,null_d,null_t,obs_t,signs,d)


#
# Pitman permutation test, data from Table 4, contrast 2
#

# Assign generic variables
x <- tbl_5$c2_three_power
y <- tbl_5$c2_five_power
z <- c(x,y)
```

```r
# Print and store observed test statistic
obs_t <- mean(x) - mean(y)
obs_t

# Compute length of all samples
n_x <- length(x)
n_y <- length(y)
n_z <- length(z)

# Generate permutation index
perm_index_x <- combinations(n_z, n_x, 1:n_z)
perm_index_y <- apply(perm_index_x, 1, vecsets::vsetdiff, x=1:n_z) %>% t()

# Generate null sample distributions
null_x <- apply(perm_index_x, 1, function(lamda) {z[lamda]}) %>% t()
null_y <- apply(perm_index_y, 1, function(lamda) {z[lamda]}) %>% t()

# Generate null test statistic distribution and compute length
null_t <- apply(null_x, 1, mean) - apply(null_y, 1, mean)
n_null <- length(null_t)

# Print components of one-sided test
n_null
sum( null_t - obs_t >= -float.tol)
( sum( null_t - obs_t >= -float.tol)/n_null ) %>% round(3)

# Clear variables
rm(n_null,n_x,n_y,n_z,null_x,null_y,null_t,obs_t,x,y,z,perm_index_x,
   perm_index_y)




# Section 3.2 -------------------------------------------------------------

#
# Wilcoxon test, data from Table 4, contrast 1
#

# Assign generic variables
d <- tbl_5$c1_five_power - tbl_5$c1_five_nopower

# Generate ranked variables
d_sign <- sign(d)
d_ranked <- rank(abs(d))
d_signedrank <- d_sign*d_ranked

# Print and store observed test statistic
obs_t <- mean(d_signedrank)
obs_t

# Compute length of all samples
n_d <- length(d_signedrank)

# Generate null sample distributions
signs <- c(-1,1)
null_signs <- permutations(2, n_d, signs, set=FALSE, repeats.allowed=TRUE)
null_d_signedrank <- sweep(null_signs,2,abs(d_signedrank),`*`)

# Generate null test statistic distribution and compute length
null_t <- apply(null_d_signedrank, 1, mean)
n_null <- length(null_t)

# Print components of two-sided test
n_null
sum( abs(null_t) - abs(obs_t) >= -float.tol)
( sum( abs(null_t) - abs(obs_t) >= -float.tol)/n_null ) %>% round(3)
```

```
# Print components of one-sided test
n_null
sum( null_t >= obs_t )
( sum( null_t >= obs_t )/n_null ) %>% round(3)

# Clear variables
rm(n_null,n_d,null_signs,null_d_signedrank,null_t,obs_t,signs,d,d_sign,
   d_ranked,d_signedrank)


#
# Wilcoxon test, data from Table 4, contrast 2
#

# Assign generic variables
d <- tbl_5$c2_three_power - tbl_5$c2_five_power

# Generate ranked variables
d_sign <- sign(d)
d_ranked <- rank(abs(d))
d_signedrank <- d_sign*d_ranked

# Print and store observed test statistic
obs_t <- mean(d_signedrank)
obs_t

# Compute length of all samples
n_d <- length(d_signedrank)

# Generate null sample distributions
signs <- c(-1,1)
null_signs <- permutations(2, n_d, signs, set=FALSE, repeats.allowed=TRUE)
null_d_signedrank <- sweep(null_signs,2,abs(d_signedrank),`*`)

# Generate null test statistic distribution and compute length
null_t <- apply(null_d_signedrank, 1, mean)
n_null <- length(null_t)

# Print components of two-sided test
n_null
sum( abs(null_t) - abs(obs_t) >= -float.tol)
( sum( abs(null_t) - abs(obs_t) >= -float.tol)/n_null ) %>% round(3)

# Print components of one-sided test
n_null
sum( null_t - obs_t >= -float.tol )
( sum( null_t - obs_t >= -float.tol )/n_null ) %>% round(3)

# Clear variables
rm(n_null,n_d,null_signs,null_d_signedrank,null_t,obs_t,signs,d,d_sign,
   d_ranked,d_signedrank)


#
# Fisher permutation test, data from Table 4, contrast 1, modified data
#

# Generate modified data
modified_tbl_5_c1_five_nopower <- tbl_5$c1_five_nopower
modified_tbl_5_c1_five_nopower[1] <- 700

# Assign generic variables
d <- tbl_5$c1_five_power - modified_tbl_5_c1_five_nopower

# Print and store observed test statistic
obs_t <- mean(d)
obs_t

# Compute length of all samples
```

```
n_d <- length(d)

# Generate null sample distributions
signs <- c(-1,1)
null_signs <- permutations(2, n_d, signs, set=FALSE, repeats.allowed=TRUE)
null_d <- sweep(null_signs,2,abs(d),`*`)

# Generate null test statistic distribution and compute length
null_t <- apply(null_d, 1, mean)
n_null <- length(null_t)

# Print components of one-sided test
n_null
sum( null_t - obs_t >= -float.tol )
( sum( null_t - obs_t >= -float.tol )/n_null ) %>% round(3)

# Clear variables
rm(null_d,null_signs,d,modified_tbl_5_c1_five_nopower,n_d,n_null,null_t,
   obs_t,signs)


#
# Wilcoxon test, data from Table 4, contrast 1, modified data
#

# Generate modified data
modified_tbl_5_c1_five_nopower <- tbl_5$c1_five_nopower
modified_tbl_5_c1_five_nopower[1] <- 700

# Assign generic variables
d <- tbl_5$c1_five_power - modified_tbl_5_c1_five_nopower

# Generate ranked variables
d_sign <- sign(d)
d_ranked <- rank(abs(d))
d_signedrank <- d_sign*d_ranked

# Print and store observed test statistic
obs_t <- mean(d_signedrank)
obs_t

# Compute length of all samples
n_d <- length(d_signedrank)

# Generate null sample distributions
signs <- c(-1,1)
null_signs <- permutations(2, n_d, signs, set=FALSE, repeats.allowed=TRUE)
null_d_signedrank <- sweep(null_signs,2,abs(d_signedrank),`*`)

# Generate null test statistic distribution and compute length
null_t <- apply(null_d_signedrank, 1, mean)
n_null <- length(null_t)

# Print components of one-sided test
n_null
sum( null_t - obs_t >= -float.tol )
( sum( null_t - obs_t >= -float.tol )/n_null ) %>% round(3)

# Clear variables
rm(n_null,n_d,null_signs,null_d_signedrank,null_t,obs_t,signs,d,d_sign,
   d_ranked,d_signedrank,modified_tbl_5_c1_five_nopower)




# Section 4.1 -------------------------------------------------------------

#
# Permutation F test, data from Table 6
```

```
#

# Assign generic variables
x <- tbl_6$levels
y <- tbl_6$earnings

# Compute length of combined samples and number of groups
n <- length(y)
n_g <- c(4,4,4)
K <- length(n_g)

# Print and store observed test statistic
obs_F <- ( lm(y ~ x) %>% anova() )$F[1]
obs_F %>% round(2)

# Generate null index and compute length
perm_index_y <- make_null_index(this_group = 1,
                                group_sizes = n_g)

# Translate null index into null sample distribution
null_y <- apply(perm_index_y, 1, function(lamda) {y[lamda]}) %>% t()
n_null <- dim(null_y)[1]

# Generate null test statistic distribution
null_F <- numeric(n_null)
for (i in 1:n_null) {
  null_F[i] <- ( lm(null_y[i,] ~ x) %>% anova() )$F[1]
}

# Print components of test
n_null
sum( null_F - obs_F >= -float.tol)
( sum( null_F - obs_F >= -float.tol)/n_null ) %>% round(3)

# Clear variables
rm(x,y,n,n_g,K,obs_F,perm_index_y,null_y,n_null,null_F,i)


#
# Pitman permutation test, data from Table 6, contrast 1
#

# Assign generic variables
x <- tbl_6$earnings[tbl_6$treatment == "UK"]
y <- tbl_6$earnings[tbl_6$treatment == "US"]
z <- c(x,y)

# Print and store observed test statistic
obs_t <- mean(x) - mean(y)
obs_t

# Compute length of all samples
n_x <- length(x)
n_y <- length(y)
n_z <- length(z)

# Generate permutation index
perm_index_x <- combinations(n_z, n_x, 1:n_z)
perm_index_y <- apply(perm_index_x, 1, vecsets::vsetdiff, x=1:n_z) %>% t()

# Generate null sample distributions
null_x <- apply(perm_index_x, 1, function(lamda) {z[lamda]}) %>% t()
null_y <- apply(perm_index_y, 1, function(lamda) {z[lamda]}) %>% t()

# Generate null test statistic distribution and compute length
null_t <- apply(null_x, 1, mean) - apply(null_y, 1, mean)
n_null <- length(null_t)

# Print components of two-sided test
```

```
n_null
sum( abs(null_t) - abs(obs_t) >= -float.tol)
( sum( abs(null_t) - abs(obs_t) >= -float.tol)/n_null ) %>% round(3)

# Print components of one-sided test
n_null
sum( null_t - obs_t >= -float.tol)
( sum( null_t - obs_t >= -float.tol)/n_null ) %>% round(3)

# Clear variables
rm(n_null,n_x,n_y,n_z,null_x,null_y,null_t,obs_t,x,y,z,perm_index_x,
   perm_index_y)




# Section 4.2 ------------------------------------------------------------

#
# Kruskal-Wallis test, data from Table SPROTT
#

# Assign generic variables
x <- tbl_6$levels
y <- tbl_6$earnings

# Compute length of combined samples and number of groups
n <- length(y)
n_g <- c(4,4,4)
K <- length(n_g)

# Print and store observed test statistic
obs_H <- kruskal.test(y,x)$statistic
obs_H %>% round(2)

# Generate null index and compute length
perm_index_y <- make_null_index(this_group = 1,
                                group_sizes = n_g)

# Translate null index into null sample distribution
null_y <- apply(perm_index_y, 1, function(lamda) {y[lamda]}) %>% t()
n_null <- dim(null_y)[1]

# Generate null test statistic distribution
null_H <- numeric(n_null)
for (i in 1:n_null) {
  null_H[i] <- kruskal.test(null_y[i,],x)$statistic
}

# Print components of test
n_null
sum( null_H - obs_H >= -float.tol)
( sum( null_H - obs_H >= -float.tol)/n_null ) %>% round(3)

# Print approximate p-value
kruskal.test(y,x)$p.value %>% round(3)

# Clear variables
rm(x,y,n,n_g,K,obs_H,perm_index_y,null_y,n_null,null_H,i)




# Section 5.1 ------------------------------------------------------------

#
# Jonckheere-Terpstra test, data from Table 7
#
```

```
# Assign generic variables
x <- tbl_7$levels
y <- tbl_7$price

# Collect and store lengths and sizes
n_g <- c(2,2,2)
K <- length(n_g)
n <- length(y)

# Define J statistic
J.fun <- function(x,y) {

  x_levels <- as.numeric(x)

  logic_mat <- sapply(x_levels ,function(e){return(e>x_levels)})
  above_mat <- sweep(logic_mat, MARGIN=2, y, `*`)
  below_mat <- sweep(logic_mat, MARGIN=1, y, `*`)

  J_mat <- above_mat > below_mat
  J <- sum(J_mat)

  return(J)

}

# Print and store observed test statistic
obs_J <- J.fun(x,y)
obs_J

# Generate null index and compute length
perm_index_y <- make_null_index(this_group = 1,
                                group_sizes = n_g)

# Translate null index into null sample distribution
null_y <- apply(perm_index_y, 1, function(lamda) {y[lamda]}) %>% t()
n_null <- dim(null_y)[1]

# Generate null test statistic distribution
null_J <- numeric(n_null)
for (i in 1:n_null) {
  null_J[i] <- J.fun(x,null_y[i,])
}

# Print components of test
n_null
sum( null_J - obs_J >= -float.tol)
( sum( null_J - obs_J >= -float.tol)/n_null ) %>% round(3)

# Clear variables
rm(null_y,i,K,n,n_g,n_null,null_J,obs_J,x,y,J.fun,perm_index_y)




# Section 5.2 -------------------------------------------------------------

#
# Directional difference test, data from Table 7
#

# Assign generic variables
x <- tbl_7$levels
y <- tbl_7$price

# Collect and store lengths and sizes
n_g <- c(2,2,2)
K <- length(n_g)
n <- length(y)
```

```
# Define J statistic
D.fun <- function(x,y) {

  x_levels <- as.numeric(x)

  logic_mat <- sapply(x_levels ,function(e){return(e>x_levels)})
  above_mat <- sweep(logic_mat, MARGIN=2, y, `*`)
  below_mat <- sweep(logic_mat, MARGIN=1, y, `*`)

  D_mat <- above_mat - below_mat
  D <- sum(D_mat)

  return(D)

}

# Print and store observed test statistic
obs_D <- D.fun(x,y)
obs_D

# Generate null index and compute length
perm_index_y <- make_null_index(this_group = 1,
                                group_sizes = n_g)

# Translate null index into null sample distribution
null_y <- apply(perm_index_y, 1, function(lamda) {y[lamda]}) %>% t()
n_null <- dim(null_y)[1]

# Generate null test statistic distribution
null_D <- numeric(n_null)
for (i in 1:n_null) {
  null_D[i] <- D.fun(x,null_y[i,])
}

# Print components of test
n_null
sum( null_D - obs_D >= -float.tol)
( sum( null_D - obs_D >= -float.tol)/n_null ) %>% round(3)

# Clear variables
rm(null_y,i,K,n,n_g,n_null,null_D,obs_D,x,y,D.fun,perm_index_y)




# Section 6.1 ------------------------------------------------------------

#
# Stratified Pitman permutation test, data from Table 8
#

# Assign generic variables
x_g1 <- tbl_8$female_25
x_g2 <- tbl_8$male_25
y_g1 <- tbl_8$female_15
y_g2 <- tbl_8$male_15

z_g1 <- c(x_g1, y_g1)
z_g2 <- c(x_g2, y_g2)

# Print and store observed test statistic
obs_t <- mean(c(x_g1,x_g2)) - mean(c(y_g1,y_g2))
obs_t

# Compute length of all samples
n_xg1 <- length(x_g1)
n_yg1 <- length(y_g1)
n_xg2 <- length(x_g2)
n_yg2 <- length(y_g2)
```

```
n_zg1 <- length(z_g1)
n_zg2 <- length(z_g2)

# Generate null sample distribution for first stratum
null_xg1 <- combinations(n_zg1, n_xg1, z_g1, set=FALSE)
null_yg1 <- apply(null_xg1, 1, vecsets::vsetdiff, x=z_g1) %>% t()
n_nullg1 <- dim(null_xg1)[1]

# Generate null sample distribution for second stratum
null_xg2 <- combinations(n_zg2, n_xg2, z_g2, set=FALSE)
null_yg2 <- apply(null_xg2, 1, vecsets::vsetdiff, x=z_g2) %>% t()
n_nullg2 <- dim(null_xg2)[1]

# Generate null sample distributions, combining strata
perm_index <- permutations(n_nullg1, 2, set=FALSE, repeats.allowed=TRUE)
null_x <- cbind( null_xg1[perm_index[,1],], null_xg2[perm_index[,2],] )
null_y <- cbind( null_yg1[perm_index[,1],], null_yg2[perm_index[,2],] )

# Generate null test statistic distribution and compute length
null_t <- apply(null_x, 1, mean) - apply(null_y, 1, mean)
n_null <- length(null_t)

# Print components of one-sided test
n_null
sum( null_t - obs_t >= -float.tol )
( sum( null_t - obs_t >= -float.tol )/n_null ) %>% round(3)

# Clear variables
rm(null_x,null_xg1,null_xg2,null_y,null_yg1,null_yg2,perm_index,n_null,n_nullg1,
   n_nullg2,n_xg1,n_xg2,n_yg1,n_yg2,n_zg1,n_zg2,null_t,obs_t,x_g1,x_g2,y_g1,
   y_g2,z_g1,z_g2)


#
# Stratified Mann-Whitney test, data from Table 8
#

# Assign generic variables
x_g1 <- tbl_8$female_25
x_g2 <- tbl_8$male_25
y_g1 <- tbl_8$female_15
y_g2 <- tbl_8$male_15

z_g1 <- c(x_g1, y_g1)
z_g2 <- c(x_g2, y_g2)
z    <- c(z_g1,z_g2)

# Compute length of all samples
n_xg1 <- length(x_g1)
n_yg1 <- length(y_g1)
n_xg2 <- length(x_g2)
n_yg2 <- length(y_g2)
n_zg1 <- length(z_g1)
n_zg2 <- length(z_g2)
n_z   <- length(z)

# Generate ranked variables
z_ranked <- rank(z)

z_g1_ranked <- z_ranked[1:n_zg1]
z_g2_ranked <- z_ranked[-(1:n_zg1)]

x_g1_ranked <- z_g1_ranked[1:n_xg1]
y_g1_ranked <- z_g1_ranked[-(1:n_xg1)]

x_g2_ranked <- z_g2_ranked[1:n_xg2]
y_g2_ranked <- z_g2_ranked[-(1:n_xg2)]

# Print and store observed test statistic
```

```
obs_t <- mean(c(x_g1_ranked,x_g2_ranked)) - mean(c(y_g1_ranked,y_g2_ranked))
obs_t

# Generate null sample distribution for first stratum
perm_index_x_g1 <- combinations(n_zg1, n_xg1, 1:n_zg1)
perm_index_y_g1 <- apply(perm_index_x_g1, 1, vecsets::vsetdiff, x=1:n_zg1) %>%
  t()

null_x_g1_ranked <- apply(perm_index_x_g1, 1, function(lamda) {z_g1_ranked[lamda]}) %>% t()
null_y_g1_ranked <- apply(perm_index_y_g1, 1, function(lamda) {z_g1_ranked[lamda]}) %>% t()

n_null_g1 <- dim(null_x_g1_ranked)[1]

# Generate null sample distribution for second stratum
perm_index_x_g2 <- combinations(n_zg2, n_xg2, 1:n_zg2)
perm_index_y_g2 <- apply(perm_index_x_g2, 1, vecsets::vsetdiff, x=1:n_zg2) %>%
  t()

null_x_g2_ranked <- apply(perm_index_x_g2, 1, function(lamda) {z_g2_ranked[lamda]}) %>% t()
null_y_g2_ranked <- apply(perm_index_y_g2, 1, function(lamda) {z_g2_ranked[lamda]}) %>% t()

n_null_g2 <- dim(null_x_g2_ranked)[1]

# Generate null sample distributions, combining strata
perm_index <- permutations(n_null_g1, 2, set=FALSE, repeats.allowed=TRUE)
null_x_ranked <- cbind( null_x_g1_ranked[perm_index[,1],],
                        null_x_g2_ranked[perm_index[,2],] )
null_y_ranked <- cbind( null_y_g1_ranked[perm_index[,1],],
                        null_y_g2_ranked[perm_index[,2],] )

# Generate null test statistic distribution and compute length
null_t <- apply(null_x_ranked, 1, mean) - apply(null_y_ranked, 1, mean)
n_null <- length(null_t)

# Print components of one-sided test
n_null
sum( null_t - obs_t >= -float.tol )
( sum( null_t - obs_t >= -float.tol )/n_null ) %>% round(3)

# Clear variables
rm(n_null,n_null_g1,n_null_g2,n_xg1,n_xg2,n_yg1,n_yg2,n_z,n_zg1,n_zg2,
   null_x_g1_ranked,null_x_g2_ranked,null_x_ranked,null_y_g1_ranked,
   null_y_g2_ranked,null_y_ranked,perm_index_x_g1,perm_index_x_g2,
   perm_index_y_g1,perm_index_y_g2,perm_index,null_t,obs_t,x_g1,x_g1_ranked,
   x_g2,x_g2_ranked,y_g1,y_g1_ranked,y_g2,y_g2_ranked,z,z_g1,z_g2,z_g1_ranked,
   z_g2_ranked,z_ranked)


#
# Pitman permutation test, data from Table 8, female only
#

# Assign generic variables
x <- tbl_8$female_25
y <- tbl_8$female_15
z <- c(x,y)

# Print and store observed test statistic
obs_t <- mean(x) - mean(y)
obs_t

# Compute length of all samples
n_x <- length(x)
n_y <- length(y)
n_z <- length(z)

# Generate permutation index
perm_index_x <- combinations(n_z, n_x, 1:n_z)
perm_index_y <- apply(perm_index_x, 1, vecsets::vsetdiff, x=1:n_z) %>% t()
```

```
# Generate null sample distributions
null_x <- apply(perm_index_x, 1, function(lamda) {z[lamda]}) %>% t()
null_y <- apply(perm_index_y, 1, function(lamda) {z[lamda]}) %>% t()

# Generate null test statistic distribution and compute length
null_t <- apply(null_x, 1, mean) - apply(null_y, 1, mean)
n_null <- length(null_t)

# Print components of two-sided test
n_null
sum( abs(null_t) - abs(obs_t) >= -float.tol)
( sum( abs(null_t) - abs(obs_t) >= -float.tol)/n_null ) %>% round(3)

# Print components of one-sided test
n_null
sum( null_t - obs_t >= -float.tol)
( sum( null_t - obs_t >= -float.tol)/n_null ) %>% round(3)

# Clear variables
rm(n_null,n_x,n_y,n_z,null_x,null_y,null_t,obs_t,x,y,z,perm_index_x,
   perm_index_y)


#
# Pitman permutation test, data from Table 8, male only
#

# Assign generic variables
x <- tbl_8$male_25
y <- tbl_8$male_15
z <- c(x,y)

# Print and store observed test statistic
obs_t <- mean(x) - mean(y)
obs_t

# Compute length of all samples
n_x <- length(x)
n_y <- length(y)
n_z <- length(z)

# Generate permutation index
perm_index_x <- combinations(n_z, n_x, 1:n_z)
perm_index_y <- apply(perm_index_x, 1, vecsets::vsetdiff, x=1:n_z) %>% t()

# Generate null sample distributions
null_x <- apply(perm_index_x, 1, function(lamda) {z[lamda]}) %>% t()
null_y <- apply(perm_index_y, 1, function(lamda) {z[lamda]}) %>% t()

# Generate null test statistic distribution and compute length
null_t <- apply(null_x, 1, mean) - apply(null_y, 1, mean)
n_null <- length(null_t)

# Print components of two-sided test
n_null
sum( abs(null_t) - abs(obs_t) >= -float.tol)
( sum( abs(null_t) - abs(obs_t) >= -float.tol)/n_null ) %>% round(3)

# Print components of one-sided test
n_null
sum( null_t - obs_t >= -float.tol)
( sum( null_t - obs_t >= -float.tol)/n_null ) %>% round(3)

# Clear variables
rm(n_null,n_x,n_y,n_z,null_x,null_y,null_t,obs_t,x,y,z,perm_index_x,
   perm_index_y)
```

```
#
# Stratified Pitman permutation test, data from Table 9
#

# Assign generic variables
x_g1 <- tbl_9$male_1x_upside
x_g2 <- tbl_9$male_5x_upside
x_g3 <- tbl_9$female_1x_upside
x_g4 <- tbl_9$female_5x_upside

y_g1 <- tbl_9$male_1x_downside
y_g2 <- tbl_9$male_5x_downside
y_g3 <- tbl_9$female_1x_downside
y_g4 <- tbl_9$female_5x_downside

# Compute length of all samples
n_x_g1 <- length(x_g1)
n_x_g2 <- length(x_g2)
n_x_g3 <- length(x_g3)
n_x_g4 <- length(x_g4)

n_z_g1 <- length(x_g1)+length(y_g1)
n_z_g2 <- length(x_g2)+length(y_g2)
n_z_g3 <- length(x_g3)+length(y_g3)
n_z_g4 <- length(x_g4)+length(y_g4)

# Print and store observed test statistic
obs_t <- sum(x_g1, x_g2, x_g3, x_g4) - sum(y_g1, y_g2, y_g3, y_g4)
obs_t

# Randomization parameters
set.seed(827205)
n_perm <- 999999
n_null <- n_perm + 1

# Generate null test statistic distribution
null_t <- numeric(n_null)
null_t[1] <- obs_t

for (i in 2:n_null) {

  this_g1 <- sample(c(x_g1,y_g1))
  this_x_g1 <- this_g1[1:n_x_g1]
  this_y_g1 <- this_g1[(n_x_g1+1):n_z_g1]

  this_g2 <- sample(c(x_g2,y_g2))
  this_x_g2 <- this_g2[1:n_x_g2]
  this_y_g2 <- this_g2[(n_x_g2+1):n_z_g2]

  this_g3 <- sample(c(x_g3,y_g3))
  this_x_g3 <- this_g3[1:n_x_g3]
  this_y_g3 <- this_g3[(n_x_g3+1):n_z_g3]

  this_g4 <- sample(c(x_g4,y_g4))
  this_x_g4 <- this_g4[1:n_x_g4]
  this_y_g4 <- this_g4[(n_x_g4+1):n_z_g4]

  this_t <- sum(this_x_g1, this_x_g2, this_x_g3, this_x_g4) -
    sum(this_y_g1, this_y_g2, this_y_g3, this_y_g4)

  null_t[i] <- this_t

}

# Print components of test
n_null
sum( abs(null_t) >= abs(obs_t) )
sum( abs(null_t) >= abs(obs_t) )/n_null
```

```
# Clear variables
rm(n_perm,n_null,n_x_g1,n_x_g2,n_x_g3,n_x_g4,n_z_g1,n_z_g2,n_z_g3,n_z_g4,
   null_t,obs_t,x_g1,x_g2,x_g3,x_g4,y_g1,y_g2,y_g3,y_g4,
   i,this_g1,this_g2,this_g3,this_g4,this_x_g1,this_x_g2,this_x_g3,
   this_x_g4, this_y_g1,this_y_g2,this_y_g3,this_y_g4,this_t)


# Section 6.2 ------------------------------------------------------------

#
# Stratified Jonckheere-Terpstra test, data from Table 7
#

# Assign generic variables
x <- tbl_7$levels
y <- tbl_7$price
s <- tbl_7$group_size

# Collect and store lengths and sizes
n_g <- c(2,2,2)
n_s <- c(1,1,1)
K <- length(n_g)
n <- length(y)

# Define J statistic
J.fun <- function(x,y) {

  x_levels <- as.numeric(x)

  logic_mat <- sapply(x_levels ,function(e){return(e>x_levels)})
  above_mat <- sweep(logic_mat, MARGIN=2, y, `*`)
  below_mat <- sweep(logic_mat, MARGIN=1, y, `*`)

  J_mat <- above_mat > below_mat
  J <- sum(J_mat)

  return(J)

}

# Print and store observed test statistic
obs_J <- J.fun(x,y)
obs_J

# Generate null index for strata (repurposed for both strata since same size)
perm_index_y <- make_null_index(this_group = 1,
                                group_sizes = n_s)

# Translate null index into null sample distributions by strata
y_s1 <- y[s==1]
null_y_s1 <- apply(perm_index_y, 1, function(lamda) {y_s1[lamda]}) %>% t()

n_null_s1 <- dim(null_y_s1)[1]

y_s2 <- y[s==2]
null_y_s2 <- apply(perm_index_y, 1, function(lamda) {y_s2[lamda]}) %>% t()

n_null_s2 <- dim(null_y_s2)[1]

# Generate null sample distributions, combining strata
perm_index <- permutations(n_null_s1, 2, set=FALSE, repeats.allowed=TRUE)
null_y_scrambled <- cbind( null_y_s1[perm_index[,1],],
                           null_y_s2[perm_index[,2],] )

# Reorder columns to match order of original data (e.g., 1,1,2,2,3,3)
null_y <- null_y_scrambled[,c(1,4,2,5,3,6)]

n_null <- dim(null_y)[1]
```

```
# Generate null test statistic distribution
null_J <- numeric(n_null)
for (i in 1:n_null) {
  null_J[i] <- J.fun(x,null_y[i,])
}

# Print components of test
n_null
sum( null_J - obs_J >= -float.tol)
( sum( null_J - obs_J >= -float.tol)/n_null ) %>% round(3)

# Clear variables
rm(null_y,i,K,n,n_g,n_null,null_J,obs_J,x,y,J.fun,perm_index_y,
   null_y_s1,null_y_s2,null_y_scrambled,perm_index,n_null_s1,
   n_null_s2,n_s,s,y_s1,y_s2)




# Section 7.1 -------------------------------------------------------------

#
# Permutation correlation test, data from Table 10, Part A
#

# Assign generic variables
x <- tbl_10$R_A
y <- tbl_10$claim_A

# Compute length of all samples
n_y <- length(y)

# Print and store observed test statistic
obs_t <- cor(x,y)
obs_t %>% round(3)

# Generate null sample distribution
null_y <- permutations(n_y, n_y, y, set=FALSE)

# Generate null test statistic distribution and compute length
null_t <- apply(null_y, 1, cor, x=x)
n_null <- length(null_t)

# Print components of one-sided test
n_null
sum( null_t - obs_t <= float.tol )
( sum( null_t - obs_t <= float.tol )/n_null ) %>% round(3)

# Clear variables
rm(null_y,n_null,n_y,null_t,obs_t,x,y)




# Section 7.2 -------------------------------------------------------------


#
# Permutation of OLS t statistic, data from Table 10,
# Model: claim_A ~ R_A
#

# Assign generic variables
y <- tbl_10$claim_A
z <- tbl_10$R_A

# Compute length of all samples
n_y <- length(y)
```

```
# Print model via OLS
fit <- lm(y~z)
fit %>% summary()

# Print and store observed test statistic
obs_t <- summary(fit)$coefficients['z','t value']
obs_t

# Generate null sample distribution
null_y <- permutations(n_y, n_y, y, set=FALSE)

# Generate null test statistic distribution and compute length
null_t <- apply(null_y, 1,
                function(this_y) summary(lm(this_y~z))$coefficients['z','t value'])
n_null <- length(null_t)

# Print components of one-sided test
n_null
sum( null_t - obs_t <= float.tol )
( sum( null_t - obs_t <= float.tol )/n_null ) %>% round(3)

# Print components of two-sided test
n_null
sum( abs(null_t) - abs(obs_t) >= -float.tol )
( sum( abs(null_t) - abs(obs_t) >= -float.tol )/n_null ) %>% round(3)

# Clear variables
rm(fit,null_y,n_null,n_y,null_t,obs_t,y,z)


#
# Permutation of OLS t statistic, data from Table new, Part B
# Model: claim_B ~ R_B
#

# Assign generic variables
y <- tbl_10$claim_B
z <- tbl_10$R_B

# Compute length of all samples
n_y <- length(y)

# Print model via OLS
fit <- lm(y~z)
fit %>% summary()

# Print and store observed test statistic
obs_t <- summary(fit)$coefficients['z','t value']
obs_t

# Generate null sample distribution
null_y <- permutations(n_y, n_y, y, set=FALSE)

# Generate null test statistic distribution and compute length
null_t <- apply(null_y, 1,
                function(this_y) summary(lm(this_y~z))$coefficients['z','t value'])
n_null <- length(null_t)

# Print components of two-sided test
n_null
sum( abs(null_t) - abs(obs_t) >= -float.tol )
( sum( abs(null_t) - abs(obs_t) >= -float.tol )/n_null ) %>% round(3)

# Clear variables
rm(fit,null_y,n_null,n_y,null_t,obs_t,y,z)


#
# Permutation of OLS t statistic, data from Table new,
```

```
# Model: claim_B ~ claim_A + R_B
#

# Assign generic variables
y <- tbl_10$claim_B
x <- tbl_10$claim_A
z <- tbl_10$R_B

# Compute length of all samples
n_y <- length(y)

# Fit model via OLS
fit <- lm(y~x+z)
summary(fit)

# Compute residuals and predictions
res_y <- lm(y~x) %>% resid()
pred_y <- lm(y~x) %>% predict()

# Print and store observed test statistic
obs_t <- summary(fit)$coefficients['z','t value']
obs_t %>% round(3)

# Generate null residual distribution
null_res_y <- permutations(n_y, n_y, res_y, set=FALSE)
null_pred_y <- matrix(
  rep(pred_y,
      dim(null_res_y)[1]
      ),
  ncol = n_y,
  byrow = TRUE
)
null_y <- null_res_y + null_pred_y

# Generate null test statistic distribution
null_t <- apply(null_y, 1,
                function(this_y) summary(lm(this_y~x+z))$coefficients['z','t value'])
n_null <- length(null_t)

# Print components of two-sided test
n_null
sum( abs(null_t) - abs(obs_t) >= -float.tol )
( sum( abs(null_t) - abs(obs_t) >= -float.tol )/n_null ) %>% round(3)

# Clear variables
rm(fit,pred_y,res_y,null_pred_y,null_res_y,null_y,n_null,n_y,null_t,obs_t,x,y,z)




#EOF
```
